# Expression of the Ebola Virus VP24 Protein Compromises the Integrity of the Nuclear Envelope and Induces a Laminopathy-Like Cellular Phenotype

**DOI:** 10.1128/mBio.00972-21

**Published:** 2021-07-06

**Authors:** Santiago Vidal, Maite Sánchez-Aparicio, Rocío Seoane, Ahmed El Motiam, Emily V. Nelson, Yanis H. Bouzaher, Maite Baz-Martínez, Isabel García-Dorival, Susana Gonzalo, Enrique Vázquez, Anxo Vidal, César Muñoz-Fontela, Adolfo García-Sastre, Carmen Rivas

**Affiliations:** a Centro de Investigación en Medicina Molecular (CIMUS), Universidade de Santiago de Compostela, Instituto de Investigaciones Sanitarias (IDIS), Santiago de Compostela, Spain; b Department of Microbiology, grid.59734.3cIcahn School of Medicine at Mount Sinai, New York, New York, USA; c Global Health and Emerging Pathogens Institute, grid.59734.3cIcahn School of Medicine at Mount Sinai, New York, New York, USA; d grid.424065.1Bernhard Nocht Institute for Tropical Medicine, Hamburg, Germany; e German Center for Infection Research (DZIF), Partner Site Hamburg, Germany; f Institute of Infection, Veterinary and Ecological Sciences, University of Liverpool, Liverpool, UK; g Department of Biochemistry and Molecular Biology, Saint Louis University, School of Medicine, Saint Louis, Missouri, USA; h Centro Nacional de Investigaciones Cardiovasculares (CNIC) Carlos III, Madrid, Spain; i Division of Infectious Diseases, Department of Medicine, grid.59734.3cIcahn School of Medicine at Mount Sinai, New York, New York, USA; j The Tisch Cancer Institute, grid.59734.3cIcahn School of Medicine at Mount Sinai, New York, New York, USA; k Centro Nacional de Biotecnología, CSIC, Cantoblanco, Madrid, Spain; Princeton University

**Keywords:** Ebola virus, laminopathies, nuclear envelope, virus-host interactions

## Abstract

Ebola virus (EBOV) VP24 protein is a nucleocapsid-associated protein that inhibits interferon (IFN) gene expression and counteracts the IFN-mediated antiviral response, preventing nuclear import of signal transducer and activator of transcription 1 (STAT1). Proteomic studies to identify additional EBOV VP24 partners have pointed to the nuclear membrane component emerin as a potential element of the VP24 cellular interactome. Here, we have further studied this interaction and its impact on cell biology. We demonstrate that VP24 interacts with emerin but also with other components of the inner nuclear membrane, such as lamin A/C and lamin B. We also show that VP24 diminishes the interaction between emerin and lamin A/C and compromises the integrity of the nuclear membrane. This disruption is associated with nuclear morphological abnormalities, activation of a DNA damage response, the phosphorylation of extracellular signal-regulated kinase (ERK), and the induction of interferon-stimulated gene 15 (ISG15). Interestingly, expression of VP24 also promoted the cytoplasmic translocation and downmodulation of barrier-to-autointegration factor (BAF), a common interactor of lamin A/C and emerin, leading to repression of the BAF-regulated *CSF1* gene. Importantly, we found that EBOV infection results in the activation of pathways associated with nuclear envelope damage, consistent with our observations in cells expressing VP24. In summary, here we demonstrate that VP24 acts at the nuclear membrane, causing morphological and functional changes in cells that recapitulate several of the hallmarks of laminopathy diseases.

## INTRODUCTION

Ebola virus (Zaire ebolavirus, EBOV) is a highly pathogenic virus that causes hemorrhagic fever with a high case fatality rate in humans. EBOV VP24 is known as the minor viral matrix protein and may have a role in nucleocapsid assembly and virus budding (1–4). In addition, VP24 inhibits type I and II interferon (IFN) signaling by both directly interacting with signal transducer and activator of transcription 1 (STAT1) and binding to karyopherin alpha (KPNA) proteins involved in nuclear import, preventing their interaction with tyrosine-phosphorylated STAT1 and therefore inhibiting its nuclear translocation (5–9). Furthermore, VP24 inhibits IFN production and diminishes the interaction between KPNA1 and heterogeneous nuclear ribonuclear protein complex C1/C2 (hnRNP C1/C2), redistributing it from the nucleus to the cytoplasm (8, 10). Interestingly, roles of VP24 in both the aberrant expression of cytokines and chemokines and dendritic cell maturation impairment by unknown mechanisms have been reported (11). The importance of VP24 during virus replication is highlighted by the fact that all the attempts to generate recombinant EBOV without VP24 have failed (12). Proteomic studies to discover novel EBOV VP24 partners have identified the nuclear membrane constituent emerin as a potential component of the VP24 cellular interactome (13, 14).

Emerin is a component of the nuclear membrane that localizes predominantly at the nuclear envelope inner membrane. Emerin belongs to the LEM (LAP2, emerin, MAN1) family of nuclear proteins. These proteins are characterized by the presence of an LEM domain (15), which consists of approximately 40 amino acids that allow them to directly bind to lamins and barrier-to-autointegration factor (BAF), a mobile lamin-binding protein that can bridge DNA and interact with histones. Heterochromatin at the nuclear periphery (16, 17) and a wide range of transcription factors (18–21) also interact with emerin. There are two types of lamin proteins, A-type (lamins A and C from the *LMNA* gene) and B-type (lamins B1 and B2 encoded by *LMNB1* and *LMNB2* genes). Lamin filaments are important for the assembly, structure, shape, and mechanical stability of metazoan nuclei but also regulate chromatin organization and gene expression and influence signaling (22, 23). Importantly, lamins, emerin, and BAF are structurally interdependent, and if any one component is missing, the other two fail to coassemble (19, 24–26). Therefore, mutations in lamin A or in lamin-binding proteins result in nuclear envelope disorganization, nuclear morphological abnormalities, accumulation of DNA damage, and an altered pattern of heterochromatin distribution and signaling abnormalities, including those affecting the mitogen-activated protein kinase (MAPK) pathway, features of many diseases collectively known as laminopathies.

The critical roles of the nuclear envelope as both a cellular barrier and a regulator of gene expression explain why many viral pathogens have evolved to modulate its permeability (27). Here, we show that EBOV VP24 interacts with emerin, lamin A, and lamin B. VP24 reduces the interaction between emerin and lamin A/C, prompts nuclear membrane disruption, and induces the activation of the DNA damage response. In addition, the expression of VP24 was associated with nuclear morphological alterations, extracellular signal-regulated kinase (ERK) pathway activation, and transcriptional changes. Finally, we demonstrate that VP24 expression leads to BAF relocation and downmodulation. In summary, here we reveal a novel activity of EBOV VP24 that results in nuclear membrane disruption and that may contribute to its critical role in virus replication and in virus pathogenesis.

## RESULTS

### VP24 interacts with emerin.

**FIG 1:**
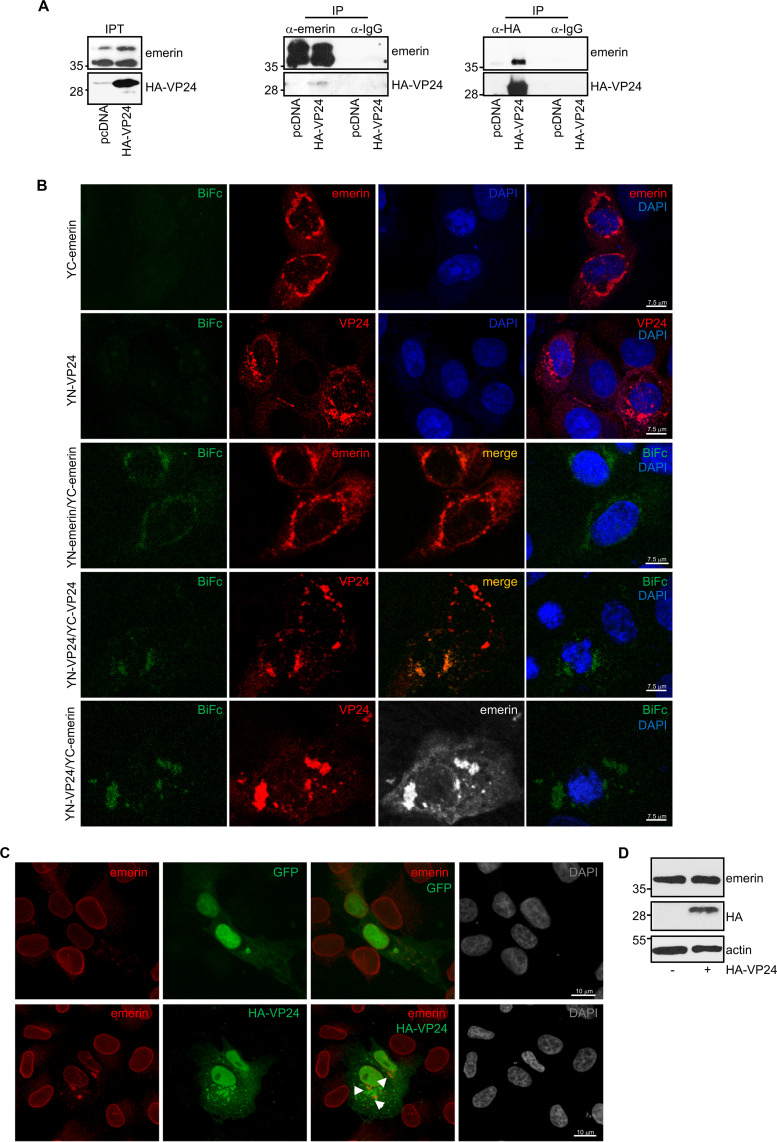
Interaction of EBOV VP24 protein with emerin. (A) Coimmunoprecipitation between VP24 and emerin. Vero cells seeded in 100-mm plates were transfected with 5 μg of pcDNA or HA-VP24, and 36 h after transfection, protein extracts of transfected cells were immunoprecipitated using anti-emerin, anti-HA, or anti-IgG antibodies. Immunoprecipitated proteins were analyzed by Western blotting using the indicated antibodies. The anti-emerin antibody detected a major band of around 37 kDa and a higher molecular weight band of around 39 kDa, probably corresponding to phosphorylated emerin. The experiments were repeated twice, and representative images of one experiment are shown; IP, immunoprecipitated samples; IPT, input cell extract. (B) VP24-emerin colocalization using the BiFc system. Vero cells were transfected with the indicated combination of the BiFc constructs (YC-emerin, C-terminal part of the yellow fluorescent protein [YFP] fused to the N terminus of full-length emerin; YN-emerin, N-terminal part of YFP fused to the N terminus of full-length emerin; YN-VP24, N-terminal part of YFP fused to the N-terminal part of full-length VP24; YC-VP24, C-terminal part of YFP fused to the N-terminal part of full-length VP24). Cells were fixed, permeabilized, and stained with anti-emerin and/or anti-VP24 primary antibodies. Chromosomes were stained with DAPI (blue). Coexpression of YC- and YN-emerin, YC- and YN-VP24, or YN-VP24 and YC-emerin led to the reconstitution of YFP signal (BiFc). The data represent more than three biological replicates. (C) Localization of endogenous emerin in Vero cells transfected with 0.3 μg of GFP or HA-VP24 or in untransfected cells. Emerin and HA-tagged VP24 are shown. Chromosomes were stained with DAPI. Arrowheads indicate colocalization of HA-VP24 and emerin. (D) Western blotting analysis using anti-emerin antibody of Vero cells at 36 h after transfection with 0.3 μg of pcDNA or HA-VP24 expression plasmids.

Two proteomic reports have identified the nuclear membrane constituent emerin as a component of the VP24 interactome (13, 14). Here, we verified the interaction between VP24 and emerin by coimmunoprecipitation assays. Vero cells were transfected with pcDNA or a hemagglutinin (HA)-tagged VP24 expression plasmid, and 36 h after transfection, immunoprecipitations were performed using anti-emerin or anti-HA antibodies. The precipitated proteins were then analyzed by Western blotting with anti-HA or anti-emerin antibodies. Coimmunoprecipitation analysis revealed that VP24 coimmunoprecipitated with emerin (Fig. 1A). To further evaluate the VP24-emerin interaction, a biomolecular fluorescence complementation (BiFc) system assay was developed as described in Materials and Methods. Vero cells cotransfected with plasmids encoding a fusion of emerin to the two complementary halves (the N-terminal end [YN] and the C-terminal end [YC]) of the yellow fluorescent protein (YFP) led to the recovery of YFP fluorescence, indicative of oligomerization of the protein, as previously demonstrated (28, 29) (Fig. 1B). Cotransfection of Vero cells with the VP24 fusion plasmids with the YN and YC halves of YFP also led to the recovery of YFP fluorescence (Fig. 1B), indicative of its oligomerization, as previously reported (2). However, a significant component of the VP24 signal was also present without reconstitution of YFP. Finally, when Vero cells were cotransfected with YN-VP24 and YC-emerin, YFP fluorescence was recovered, indicative of the emerin-VP24 interaction (Fig. 1B). We then evaluated whether EBOV VP24 colocalizes with endogenous emerin. Vero cells were transfected with green fluorescent protein (GFP) or HA-VP24, and 36 h after transfection, immunostaining of HA-VP24 and endogenous emerin was carried out. VP24 protein was detected both in the nucleus and cytoplasm of transfected cells, as reported previously (30). Whereas endogenous emerin was mainly localized to the nuclear rim in GFP-transfected cells (Fig. 1C), it was observed lining the nuclear membrane and in cytoplasmic aggregates in HA-VP24-expressing cells (Fig. 1C and Fig. S1 in the supplemental material). We observed partial colocalization between VP24 and emerin (Fig. 1C and Fig. S1). Western blotting revealed that the overall level of emerin was identical independent of the expression of VP24 (Fig. 1D).

10.1128/mBio.00972-21.1 FIG S1 Partial colocalization between transfected VP24 and endogenous emerin. Localization of endogenous emerin in Vero cells transfected with 0.3 μg of GFP or HA-VP24. Emerin and HA-tagged VP24 are shown. Chromosomes were stained with DAPI. Arrowheads indicate colocalization of HA-VP24 and emerin. Download FIG S1, PDF file, 0.1 MB. Copyright © 2021 Vidal et al. 2021 Vidal et al. https://creativecommons.org/licenses/by/4.0/ This content is distributed under the terms of the Creative Commons Attribution 4.0 International license.

### VP24 interacts with both lamin A and lamin B.

**FIG 2:**
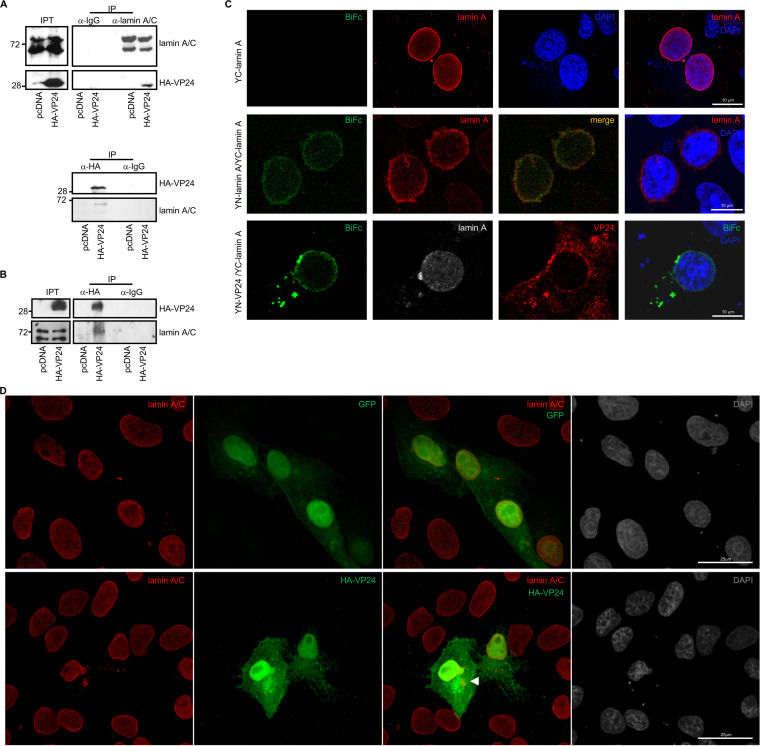
VP24 interacts with lamin A/C. (A) Coimmunoprecipitation between VP24 and lamin A/C. Vero cells were transfected with 5 μg of HA-VP24 in a 100-mm dish, and 36 h after transfection, protein extracts of transfected cells were immunoprecipitated using anti-HA, anti-lamin A/C, or anti-IgG antibodies. Immunoprecipitated proteins were analyzed by Western blotting using the indicated antibodies. The experiments were repeated twice, and representative images of one experiment are shown; IP, immunoprecipitated samples; IPT, input cell extract. (B) HUH-7 cells were transfected with 5 μg of HA-VP24 in a 100-mm dish, and 36 h after transfection, protein extracts of transfected cells were immunoprecipitated using anti-HA antibody. Immunoprecipitated proteins were analyzed by Western blotting using anti-lamin A/C antibody; IP, immunoprecipitated samples; IPT, input cell extract. (C) VP24-lamin A colocalization using the BiFc system. Vero cells were transfected with the indicated combination of the BiFc constructs (YC-lamin A, C-terminal part of the yellow fluorescent protein [YFP] fused to the N terminus of full-length lamin A; YN-lamin A, N-terminal part of YFP fused to the N terminus of full-length lamin A; YN-VP24, N-terminal part of YFP fused to the N-terminal part of full-length VP24). Cells were fixed, permeabilized, and stained with anti-lamin A/C and/or anti-VP24 primary antibodies. Chromosomes were stained with DAPI (blue). Coexpression of YC- and YN-lamin or YN-VP24 and YC-lamin A led to the reconstitution of YFP signal (BiFc). The data represent more than three biological replicates. Chromosomes were stained with DAPI (blue). (D) Localization of lamin A/C in Vero cells transfected with 0.3 μg of GFP or HA-VP24 or in untransfected cells. Lamin A/C and HA-tagged VP24 are shown. Chromosomes were stained with DAPI. Arrowhead indicates colocalization of HA-VP24 and lamin A/C.

Emerin localization at the nuclear envelope has been shown to depend on lamin A (31–35). Therefore, we decided to study the putative interaction between transfected HA-VP24 and endogenous lamin A/C protein by coimmunoprecipitation assays. Vero cells were transfected with pcDNA or HA-VP24, and 36 h after transfection, immunoprecipitations were performed using anti-lamin A/C or anti-HA antibodies. The precipitated proteins were then analyzed by Western blotting with anti-HA or anti-lamin A/C antibodies. We observed coimmunoprecipitation between lamin A/C and VP24 (Fig. 2A). Similar experiments carried out in HUH-7 cells revealed that lamin A/C also coimmunoprecipitated with VP24 when expressed in these cells (Fig. 2B). Interaction between VP24 and lamin A was also evaluated using a BiFc system assay. Vero cells cotransfected with the plasmids encoding a fusion of lamin A to the two complementary halves of YFP led to the recovery of YFP fluorescence, indicative of oligomerization of the protein, as previously demonstrated (36) (Fig. 2C). We also observed reconstitution of YFP fluorescence in Vero cells cotransfected with YC-lamin A and YN-VP24 plasmids, indicative of an interaction between lamin A and VP24 (Fig. 2C). Colocalization between endogenous lamin A/C and HA-VP24 was then analyzed by immunofluorescence staining and confocal analysis. Vero cells were transfected with GFP or HA-VP24, and 36 h after transfection, cells were fixed and immunostained with anti-lamin A/C and anti-HA antibodies. Endogenous lamin A/C was detected as a nuclear rim stain, indicating its location at the nuclear envelope in cells expressing or not expressing HA-VP24 (Fig. 2D and Fig. S2). Colocalization between VP24 and lamin A was detected both at the nuclear membrane as well as in some lamin A cytoplasmic aggregates, which were only present in VP24-expressing cells (Fig. 2D and Fig. S2).

10.1128/mBio.00972-21.2 FIG S2 Partial colocalization between transfected VP24 and endogenous lamin A/C. Localization of endogenous lamin A/C in Vero cells transfected with 0.3 μg of GFP or HA-VP24. Lamin A/C and HA-tagged VP24 are shown. Chromosomes were stained with DAPI. Arrowheads indicate colocalization of HA-VP24 and lamin A/C. Download FIG S2, PDF file, 0.4 MB. Copyright © 2021 Vidal et al. 2021 Vidal et al. https://creativecommons.org/licenses/by/4.0/ This content is distributed under the terms of the Creative Commons Attribution 4.0 International license.

**FIG 3:**
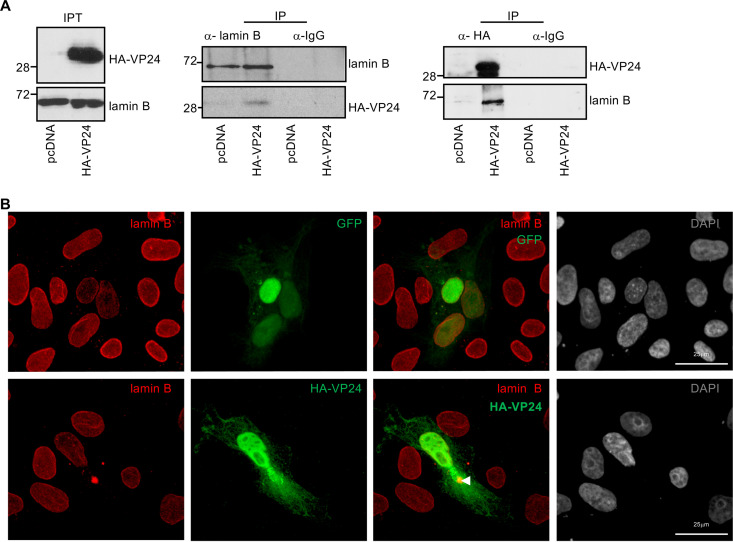
VP24 interacts with lamin B. (A) Coimmunoprecipitation between VP24 and lamin B. Vero cells were transfected with 5 μg of HA-VP24 in a 100-mm dish, and 36 h after transfection, protein extracts of transfected cells were immunoprecipitated using anti-lamin B, anti-HA, or anti-IgG antibodies. Immunoprecipitated proteins were analyzed by Western blotting using the indicated antibodies. The experiments were repeated twice, and representative images of one experiment are shown; IP, immunoprecipitated samples; IPT, input cell extract. (B) Localization of lamin B in Vero cells transfected with 0.3 μg of GFP or HA-VP24 or in untransfected cells. Lamin B and HA-tagged VP24 are shown. Chromosomes were stained with DAPI. Arrowhead indicates colocalization of HA-VP24 and lamin B.

Emerin also binds B-type lamins (32, 34). In addition, lamin B has been identified as a component of the VP24 interactome in a proteomic study (13). Therefore, we investigated the interaction between endogenous lamin B and HA-VP24. Vero cells were transfected with pcDNA or HA-VP24, and 36 h after transfection, immunoprecipitations were performed using anti-lamin B or anti-HA antibodies. The precipitated proteins were then analyzed by Western blotting with anti-HA or anti-lamin B antibodies. Coimmunoprecipitation analysis revealed that both proteins interact (Fig. 3A). We next analyzed the colocalization of endogenous lamin B with HA-VP24. Vero cells were transfected with GFP or HA-VP24, and 36 h after transfection, cells were fixed and immunostained with anti-lamin B and anti-HA antibodies. Endogenous lamin B was detected mainly at the nuclear envelope (Fig. 3B and Fig. S3). Colocalization between VP24 and lamin B was detected both at the nuclear rim as well as in some lamin B cytoplasmic aggregates (Fig. 3B and Fig. S3). Altogether, these data indicate that VP24 interacts with both lamin A/C and B, and VP24 appears to displace a fraction of these proteins from the nuclear envelope to the cytoplasm.

10.1128/mBio.00972-21.3 FIG S3 Partial colocalization between transfected VP24 and endogenous lamin B. Localization of endogenous lamin B in Vero cells transfected with 0.3 μg of GFP or HA-VP24. Lamin B and HA-tagged VP24 are shown. Chromosomes were stained with DAPI. Arrowheads indicate colocalization of HA-VP24 and lamin B. Download FIG S3, PDF file, 3.0 MB. Copyright © 2021 Vidal et al. 2021 Vidal et al. https://creativecommons.org/licenses/by/4.0/ This content is distributed under the terms of the Creative Commons Attribution 4.0 International license.

### VP24 interacts with emerin in a tag-independent manner.

**FIG 4:**
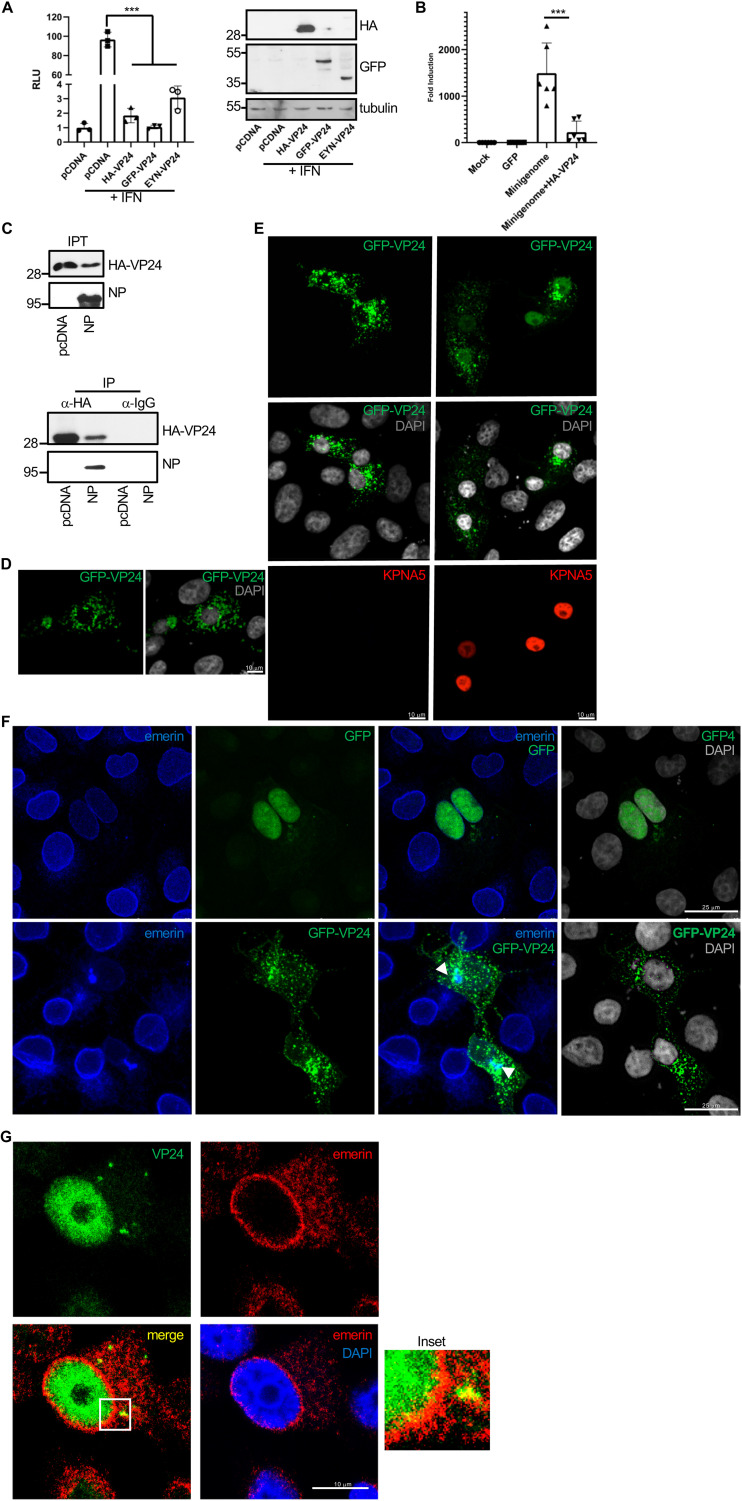
EBOV VP24 protein interacts with emerin in a tag-independent manner. (A) HEK-293 cells were cotransfected with ISG54-luciferase together with pcDNA or the indicated VP24 expression plasmids. At 24 h after transfection, cells were treated with IFN-α, and at 16 h after treatment, luciferase production was analyzed. Columns are representative of the mean, and error bars represent the standard deviation of three biological replicates (left). Cell lysates from the experiment were analyzed by Western blotting for VP24 expression (right). (B) Minigenome assay in cells cotransfected with HA-VP24. Columns are representative of the mean, and error bars represent the standard deviation of three biological replicates. (C) Coimmunoprecipitation between HA-VP24 and NP. Vero cells were cotransfected with 2.5 μg of HA-VP24 and 2.5 μg of pcDNA or NP expression plasmids in a 100-mm dish, and 36 h after transfection, protein extracts of transfected cells were immunoprecipitated using anti-HA antibody. Immunoprecipitated proteins were analyzed by Western blotting using the indicated antibodies. The experiments were repeated twice, and representative images of one experiment are shown; IP, immunoprecipitated samples; IPT, input cell extract. (D) Localization of GFP-VP24 protein in Vero cells. Chromosomes were stained with DAPI. (E) Localization of GFP-VP24 protein in cells cotransfected with pcDNA or karyopherin 5 (KPNA5) expression plasmid. Chromosomes were stained with DAPI. (F) Colocalization of emerin and GFP-VP24 in Vero cells cotransfected with GFP or GFP-VP24. Chromosomes are stained with DAPI. Arrowheads indicate colocalization of GFP-VP24 and emerin. (G) Colocalization of emerin and VP24 in Vero cells transfected with untagged VP24 expression plasmid. Chromosomes are stained with DAPI. Inset shows higher magnification of the boxed area.

Tagging proteins may alter subcellular localization, stability, activity, or interaction with binding partners. Thus, fusion of VP24 with a Flag-tag at its C terminus inhibits the ability of VP24 to form nucleocapsid-like structures and inhibits transcription and replication of the EBOV genome (37). Therefore, we decided to evaluate whether the HA-tagged VP24 protein employed in this study shares the main characteristics previously described for the viral protein. First, we analyzed the ability of HA-VP24 to inhibit IFN signaling. HEK-293 cells were cotransfected with ISG54-luciferase together with beta-galactosidase and pcDNA, HA-VP24, YN-VP24, or a GFP-tagged VP24 construct, and 24h after transfection, cells were treated with IFN-α for 16 h. Cell extracts were then harvested and analyzed for luciferase and beta-galactosidase activities. As shown in Figure 4A, IFN induces transactivation of the reporter, as expected, and all VP24 constructs significantly reduced this transactivation, indicating their ability to inhibit IFN signaling. Then, we evaluated the ability of HA-VP24 to modulate luciferase expression from an EBOV minigenome. HEK-293 cells were cotransfected with plasmids for the expression of the T7 polymerase, nucleoprotein (NP), VP30, VP35, and L protein of the Zaire EBOV minigenome strain Mayinga, as previously described (38), together with pcDNA or HA-VP24. At 48 h after transfection, luciferase activity was analyzed. Transfection of HA-VP24 inhibited luciferase expression from the minigenome, as previously reported (37, 39–41) (Fig. 4B). Another function of VP24 is its ability to interact with NP to facilitate nucleocapsid assembly and genome packaging (42). We then evaluated the coimmunoprecipitation between HA-VP24 and NP. We cotransfected Vero cells with HA-VP24 together with the NP expression plasmid or the empty vector pcDNA. At 36 h after transfection, immunoprecipitation was performed using anti-HA antibody. The precipitated proteins were then analyzed by Western blotting with anti-HA or anti-NP antibodies. We observed coimmunoprecipitation between NP and VP24 (Fig. 4C). Altogether, these results indicated that the fusion of HA-tag to the N terminus of VP24 does not alter the main functions of the viral protein.

However, one feature of the HA-VP24 protein is not consistent with some previous VP24 descriptions. Ebola VP24 protein has been initially described as a cytoplasmic protein in both infected and transfected cells (2), similar to the subcellular localization of the protein fused to YC or YN of YFP, as shown in Figure 1B. However, HA-VP24 protein was detected both in the nucleus and cytoplasm of Vero cells, as recently reported (30). The small size of the VP24 protein could facilitate a passive cytoplasm-to-nucleus translocation of the protein. Cytoplasmic proteins with a molecular mass larger than 40 to 45 kDa are unable to enter the nucleus unless they provide a signal for nuclear import (43). Therefore, we decided to evaluate the subcellular localization of the VP24 protein after fusing to a 30-kDa GFP tag. As shown in Figure 4D, confocal analysis revealed that GFP-VP24 protein was detected mainly at the cell cytoplasm, suggesting that HA-VP24 protein entered into the nucleus by passive diffusion. Although VP24 does not have a classical targeting signal to go inside the nucleus, it interacts with members of the importin superfamily of nuclear import transporters (6). To evaluate whether this interaction can facilitate the entry of the viral protein inside the nucleus, we analyzed the localization of GFP-VP24 protein after karyopherin overexpression. Confocal analysis showed that GFP-VP24 protein localized in the nucleus of cells overexpressing karyopherin (Fig. 4E), suggesting that the interaction with the nuclear import transporter may promote its nuclear translocation. We then decided to evaluate whether the mainly cytoplasmic GFP-VP24 protein was still able to colocalize with emerin. Vero cells were transfected with GFP-VP24, and 36 h after transfection, cells were fixed and immunostained with anti-emerin antibody. A fraction of endogenous emerin was detected in cytoplasmic aggregates, partially colocalizing with GFP-VP24 protein (Fig. 4F), indicating the interaction of GFP-VP24 and emerin. Finally, we decided to evaluate the colocalization between untagged VP24 protein and emerin. BSR-T7 cells were transfected with a plasmid encoding untagged VP24, and 36 h after transfection, cells were stained with anti-VP24 and anti-emerin antibodies. VP24 was detected both at the nucleus and in cytoplasmic aggregates, and a partial colocalization with emerin was observed (Fig. 4G). Altogether, these results indicate that VP24 interacts with emerin in a tag-independent manner.

### VP24 associates with emerin in the presence of other EBOV proteins.

**FIG 5:**
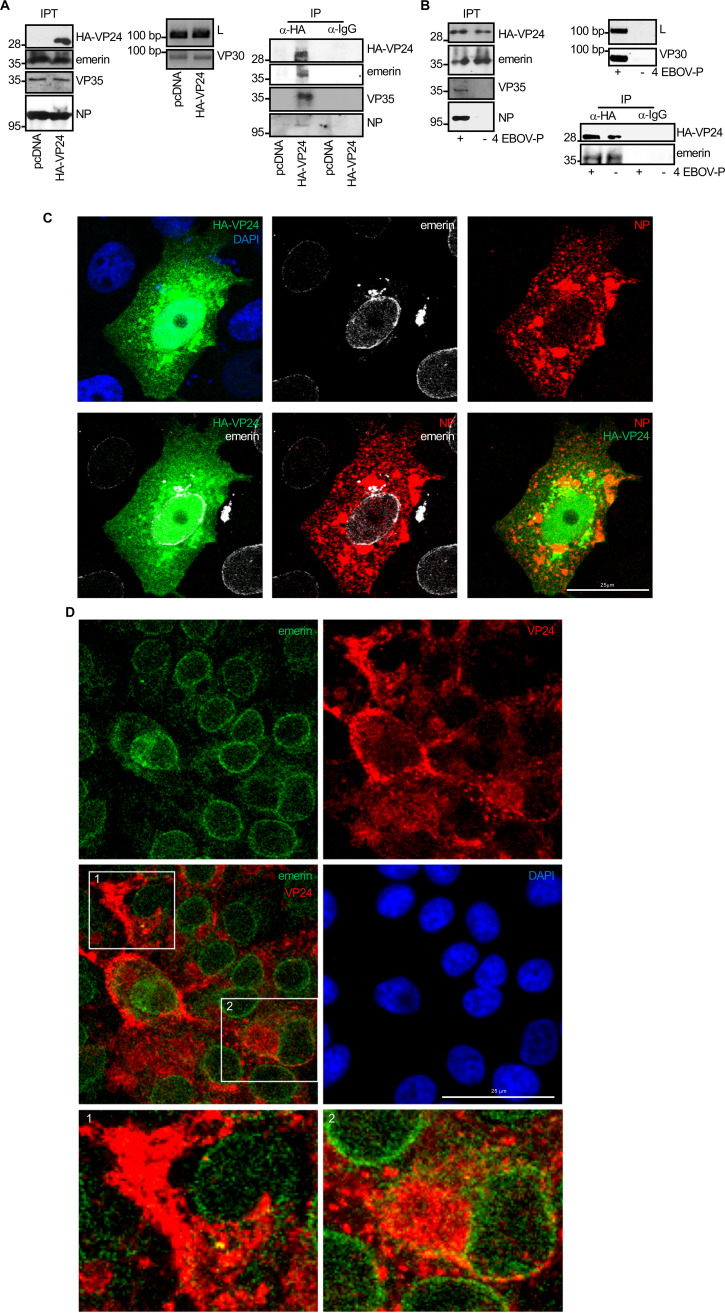
VP24 associates with emerin in the presence of other EBOV proteins. (A) Coimmunoprecipitation between VP24 and emerin in BSR-T7 cells cotransfected with the plasmids from the EBOV minigenome, NP, L, VP30, and VP35 (strain Makona), and in the presence or absence of HA-VP24. Expression of VP35, NP, and HA-VP24 was confirmed by Western blotting with the indicated antibodies. Expression of VP30 and L protein was verified by reverse-transcription-PCR (RT-PCR). Coimmunoprecipitation of both VP35 and NP with HA-VP24 was also confirmed; IP, immunoprecipitated samples; IPT, input cell extract. (B) Coimmunoprecipitation between VP24 and emerin in BSR-T7 cells cotransfected with HA-VP24 and pcDNA or the plasmids from the EBOV minigenome NP, L, VP30, and VP35 (strain Makona) (4 EBOV-P). Expression of VP35, NP, and HA-VP24 was confirmed by Western blotting with the indicated antibodies. Expression of VP30 and L protein was verified by RT-PCR; IP, immunoprecipitated samples; IPT, input cell extract. (C) Colocalization of HA-VP24 and emerin in Vero cells cotransfected with HA-VP24 and NP. Emerin, NP, and HA-VP24 localization are shown. Chromosomes were stained with DAPI. (D) Colocalization of VP24 and emerin in Vero cells infected with EBOV. Emerin and VP24 localization are shown. Chromosomes were stained with DAPI. Images 1 and 2 show higher magnification views of the indicated boxed areas.

Our results indicate that VP24 protein, in the absence of other EBOV proteins, interacts with emerin. Previous studies have shown that VP24 colocalizes with NP in a time-dependent manner in the course of EBOV infection in Vero cells (40). Therefore, we decided to evaluate whether HA-VP24 protein expressed together with EBOV proteins involved in RNA replication still interacted with emerin. We cotransfected 1 × 10^5^ BSR-T7 cells with the expression plasmids for the EBOV Makona proteins NP (0.059 μg), VP35 (0.029 μg), VP30 (0.029 μg), and L (0.029 μg), and in the presence or absence of HA-VP24 (0.041 μg). At 36 h after transfection, immunoprecipitations were performed using anti-HA antibody. The precipitated proteins were then analyzed by Western blotting with antibodies against HA-tag, emerin, or the EBOV proteins NP and VP35. We observed that HA-VP24 coimmunoprecipitated with NP and VP35. Moreover, we also observed that HA-VP24 coimmunoprecipitated with emerin when coexpressed with EBOV NP, VP35, VP30, and L (Fig. 5A). In addition, coimmunoprecipitation analysis between HA-VP24 and emerin in cells cotransfected with HA-VP24 and pcDNA or the EBOV NP, VP35, VP30, and L expression plasmids did not reveal differences in the VP24-emerin interaction (Fig. 5B). We also analyzed the putative colocalization of VP24 and emerin in the presence of NP. Vero cells were cotransfected with HA-VP24 and NP expression plasmids, and 36 h after transfection, we fixed the cells and immunostained using anti-HA, anti-emerin, and anti-NP antibodies. NP was detected in cytoplasmic inclusions, whereas HA-VP24 localized both in the nucleus and in some cytoplasmic aggregates where it partially colocalized with NP (Fig. 5C and Fig. S4). Emerin was detected at the nuclear rim and in some cytoplasmic aggregates, and a partial colocalization with VP24 was observed (Fig. 5C and Fig. S4), indicating that VP24 can associate with emerin in the presence of NP. Finally, we evaluated the putative VP24-emerin colocalization in Vero cells infected with EBOV. Emerin was observed lining the nuclear membrane, diffused in the cytoplasm, and in some small cytoplasmic aggregates. Colocalization between emerin and VP24 was observed both at the nuclear membrane as well as in cytoplasmic aggregates (Fig. 5D). Altogether, these results indicate that VP24 interacts with emerin independent of the expression of other EBOV proteins.

10.1128/mBio.00972-21.4 FIG S4 Partial colocalization between transfected VP24 and endogenous emerin in the presence of EBOV NP. Colocalization of HA-VP24 and emerin in Vero cells cotransfected with HA-VP24 and NP. Emerin, NP, and HA-VP24 localizations are shown. Download FIG S4, PDF file, 0.3 MB. Copyright © 2021 Vidal et al. 2021 Vidal et al. https://creativecommons.org/licenses/by/4.0/ This content is distributed under the terms of the Creative Commons Attribution 4.0 International license.

### Loss of nuclear membrane integrity upon EBOV VP24 expression.

**FIG 6:**
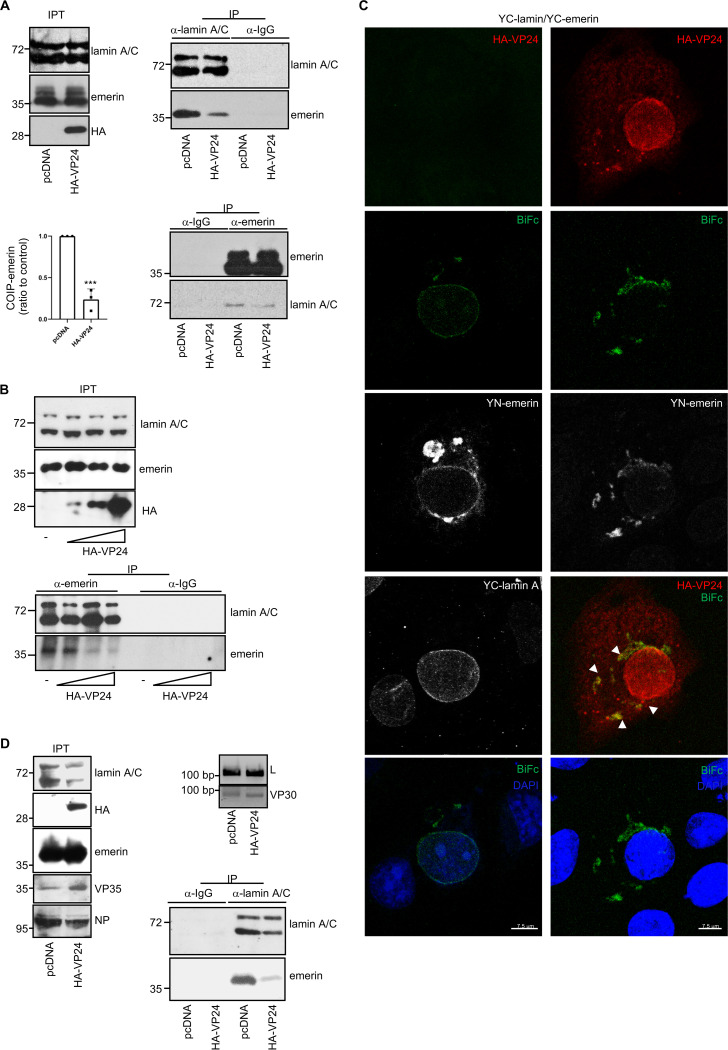
VP24 reduces the interaction between emerin and lamin A/C. (A) Coimmunoprecipitation between emerin and lamin A/C in nontransfected cells and in cells transfected with 2.5 μg of HA-VP24 in a 100-mm dish. Representative blots of one experiment are shown. Results obtained from three independent coimmunoprecipitation experiments were quantified. The amount of coimmunoprecipitated emerin was normalized to the amount of lamin A/C protein immunoprecipitated in each experiment; IP, immunoprecipitated samples; IPT, input cell extract. The emerin/lamin detected in the pcDNA-transfected cells was set to 1, and the values found in VP24-expressing cells were calculated relative to it. Statistical analysis was assessed by a Student’s *t* test. ***, *P* < 0.001. (B) Coimmunoprecipitation between emerin and lamin A/C in cells transfected with different amounts (0, 1.2, 2.5, or 5 μg per 100-mm dish) of HA-VP24 expression plasmid. IP, immunoprecipitated samples; IPT, input cell extract. (C) Emerin-lamin A colocalization using the BiFc system in cells expressing VP24. Chromosomes were stained with DAPI (blue). Arrowheads indicate colocalization of V24 with the BiFc signal. (D) Coimmunoprecipitation between emerin and lamin A/C in BSR-T7 cells cotransfected with the plasmids L, NP, VP30, and VP35 of the EBOV minigenome system and in presence or absence or HA-VP24. Expression of VP35, NP, and HA-VP24 was confirmed by Western blotting with the indicated antibodies. Expression of VP30 and L protein was verified by reverse-transcription-PCR (RT-PCR). Representative blots of one experiment are shown; IP, immunoprecipitated samples; IPT, input cell extract.

Mutations in nuclear envelope components blocking the interaction between emerin and lamins are associated with the mislocalization of nuclear envelope components (44–46). We then decided to evaluate whether VP24 modulates the interaction between emerin and lamin A/C. First, we analyzed the coimmunoprecipitation between emerin and lamin A/C in cells expressing VP24. HEK-293 cells transfected with HA-VP24 or pcDNA were subjected to immunoprecipitation with anti-emerin or anti-lamin A/C antibodies. Immunoprecipitated proteins were then analyzed by Western blotting with the same antibodies. As shown in Figure 6A, emerin coimmunoprecipitated with lamin A/C in pcDNA-transfected cells, as expected. We observed a clear reduction in the coimmunoprecipitation between both proteins in those cells expressing VP24 (Fig. 6A). Quantification of the emerin protein that coimmunoprecipitated with lamin A/C in three independent experiments revealed that expression of VP24 significantly reduced the amount of emerin interacting with lamin A/C (*P* < 0.001; Fig. 6A). Additionally, we evaluated the emerin-lamin A/C coimmunoprecipitation in HEK-293 cells transfected with different doses (0, 1.25, 2.5, or 5 μg per 100-mm dish) of HA-VP24 expression vector. A clear reduction in the coimmunoprecipitation between both proteins was observed in the cells transfected with the two highest doses of HA-VP24 (Fig. 6B). To further evaluate the consequences of VP24 expression on emerin-lamin interaction, we analyzed the emerin/lamin A complexes using the BiFc system in cells expressing VP24. Confocal analysis revealed that cells cotransfected with YN-emerin and YC-lamin A showed a fluorescent signal mainly at the nuclear rim, indicating the interaction of emerin and lamin A at the inner nuclear membrane, as expected. An uneven fluorescent signal located both at the nuclear envelope and in some cytoplasmic aggregates was observed in the VP24-expressing cells (Fig. 6C and Fig. S5), indicating that the remaining interactions between emerin and lamin A are not happening at the right compartment, the nuclear rim. An association between the BiFc signal and VP24 cytoplasmic protein was also observed (Fig. 6C and Fig. S5). Finally, we decided to evaluate whether the expression of VP24 in the context of the EBOV replicative proteins also altered the emerin-lamin A/C interaction. We cotransfected 1 × 10^5^ BSR-T7 cells with the expression plasmids for the EBOV Makona proteins NP (0.059 μg), VP35 (0.029 μg), VP30 (0.029 μg), and L (0.029 μg) and in presence or absence of HA-VP24 (0.041 μg). At 36 h after transfection, immunoprecipitations were performed using anti-lamin antibody. The precipitated proteins were then analyzed by Western blotting with anti-lamin A/C or anti-emerin antibodies. We observed a clear reduction in the coimmunoprecipitation between both proteins in those cells expressing VP24 (Fig. 6D).

10.1128/mBio.00972-21.5 FIG S5 VP24 reduces the colocalization between emerin and lamin A/C. Emerin-lamin A colocalization using the BiFc system in cells expressing or not expressing HA-VP24. Chromosomes were stained with DAPI (blue). Arrowheads indicate colocalization of VP24 with the BiFc signal. Download FIG S5, PDF file, 1.8 MB. Copyright © 2021 Vidal et al. 2021 Vidal et al. https://creativecommons.org/licenses/by/4.0/ This content is distributed under the terms of the Creative Commons Attribution 4.0 International license.

**FIG 7:**
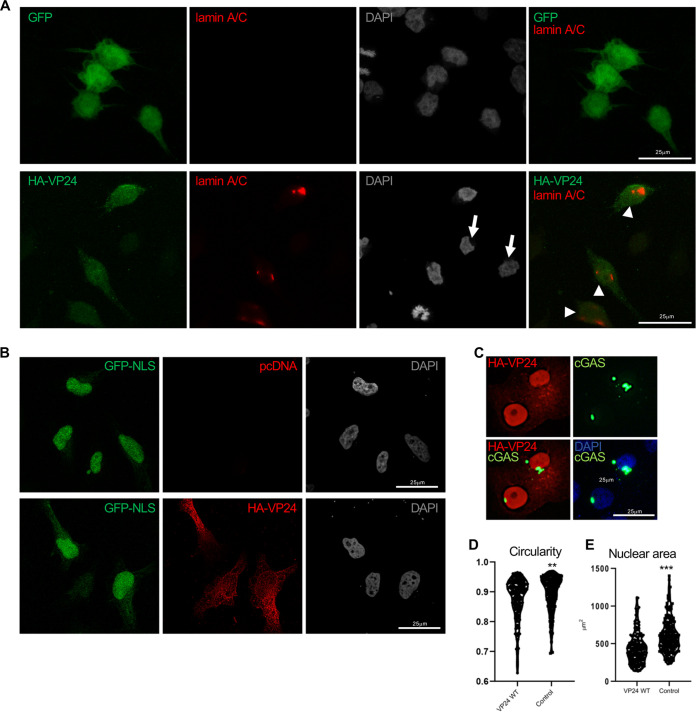
VP24 induces nuclear membrane disruption. (A) Immunofluorescence staining using anti-lamin A/C antibody of cells transfected with 0.3 μg of GFP or VP24 and permeabilized with digitonin. Chromosomes were stained with DAPI. Arrowheads indicate positive detection of lamin A/C in cells expressing VP24, and arrows indicate untransfected cells. (B) Localization of GFP-NLS in cells cotransfected with 0.3 μg of pcDNA or HA-VP24 and permeabilized with digitonin. Chromosomes were stained with DAPI. (C) Localization of GFP-cGAS in Vero cells transfected with 0.3 μg of HA-VP24. (D) Shape of the nucleus of Vero cells expressing HA-VP24 or control cells. A higher circularity denotes a more circular shape. (E) Size of the nucleus of Vero cells expressing HA-VP24 or control cells. Graphs show one data point per nucleus analyzed. Statistical analysis was assessed by a Student’s *t* test. **, *P* < 0.01; ***, *P* < 0.001.

Since nuclear envelope disorganization triggers its collapse (47), we hypothesized that VP24 expression could lead to nuclear envelope damage. To address this hypothesis, we carried out immunofluorescence analysis using anti-lamin A/C antibody of cells transfected with HA-VP24 after permeabilization with digitonin, a compound that permeabilizes the plasma membrane but leaves the nuclear envelope intact (48, 49). Consequently, antibodies to lamin A/C, located at the inner nuclear membrane, can bind their antigens in digitonin-permeabilized cells only if nuclear membranes are damaged (50). As shown in Figure 7A, lamin A/C was virtually undetectable in digitonin-treated GFP-transfected or untransfected cells; however, a lamin A/C signal was clearly observed in those cells expressing VP24 (Fig. 7A), suggesting that the nuclear membrane is damaged. To ascertain whether nuclear membrane was damaged by VP24 protein, we analyzed the distribution of the fluorescent nuclear envelope rupture reporter GFP-nuclear localization signal (NLS) in cells expressing VP24. GFP-NLS localized to the nucleus in cells cotransfected with pcDNA, whereas it localized to the nucleus but also spilled into the cytoplasm in those cells expressing VP24 (Fig. 7B). Finally, we analyzed the subcellular localization of another fluorescent nuclear envelope rupture reporter, GFP-cyclic GMP-AMP synthase (cGAS). After nuclear membrane breakdown, the GFP-cGAS located at the cytoplasm has been reported to bind to exposed genomic DNA and accumulate at the break site (51). As shown in Figure 7C, GFP-cGAS was detected in discrete intranuclear foci in cells expressing VP24. Altogether, these results indicate that VP24 expression induces the loss of nuclear membrane integrity.

Nuclear shape is linked to the structure of the lamina (52). Therefore, we analyzed the shape and area of the nucleus of cells expressing VP24. Whereas nuclei of control cells are roughly circular or slightly ovoid, those cells expressing VP24 are often irregular (Fig. 7D). We also observed that the median nuclear size of cells transfected with VP24 was significantly smaller than that observed in control cells (Fig. 7E). Altogether, these results indicated that expression of VP24 altered the nuclear morphology of the cells.

### Activation of MAPK pathways.

**FIG 8:**
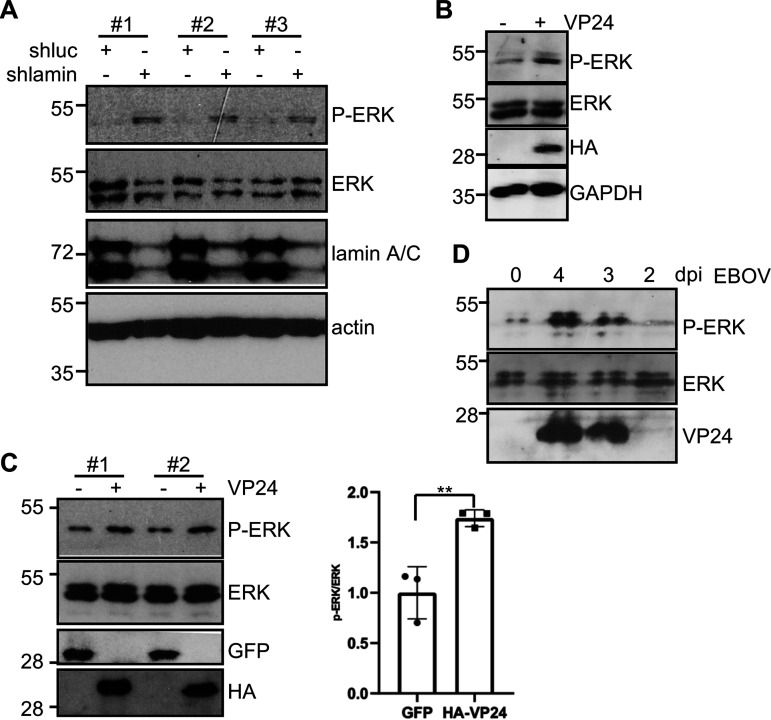
VP24 protein activates ERK1/2. (A) HeLa cells stably transfected with short hairpin RNA specific to luciferase (shluc) or short hairpin RNA specific to lamin A (shlamin A) plasmids were analyzed by Western blotting with the indicated antibodies. Three replicates are shown. (B) Vero cells were transfected with pcDNA or HA-VP24, and 36 h after transfection, cells were tested for the presence of phosphorylated ERK1/2 (P-ERK) and total ERK1/2, as indicated. (C) HeLa cells were transfected with the indicated plasmids, and 36 h after transfection, cells were tested for the presence of phosphorylated ERK1/2 and total ERK1/2, as indicated. Results from two different transfections are shown (left). Phospho-ERK and ERK protein intensity bands from three biological replicates were quantified using ImageJ software. The P-ERK/ERK ratios from each respective time were plotted. Data represent the mean and error bars of 3 biological replicates (right). Statistical analysis was assessed by a Student’s *t* test. **, *P* < 0.01. (D) Western blotting analysis with anti-phospho-ERK, anti-ERK, and anti-VP24 antibodies in Vero cells at different times after infection with EBOV. The 0 time corresponds to mock-infected cells that were in culture for 4 days.

Nuclear envelope disorganization detected in laminopathies has been shown to stimulate the phosphorylation of ERK (53–55). In addition, downmodulation of lamin A/C also induces ERK phosphorylation (Fig. 8A). To determine whether expression of VP24 also activates MAPK signaling, we measured phosphorylation of ERK1/2 (pERK1/2) in transiently transfected Vero cells expressing VP24. We observed that the expression of VP24 significantly increased the amount of phosphorylated protein, indicating that VP24 activates MAPK cascades (Fig. 8B). Increased ERK1/2 phosphorylation was also observed in HeLa cells after transfection with VP24 expression plasmid (Fig. 8C). Interestingly, we also observed induction of ERK phosphorylation in response to infection with authentic EBOV (Fig. 8D).

### Induction of DNA damage and upregulation of ISG15 by VP24.

**FIG 9:**
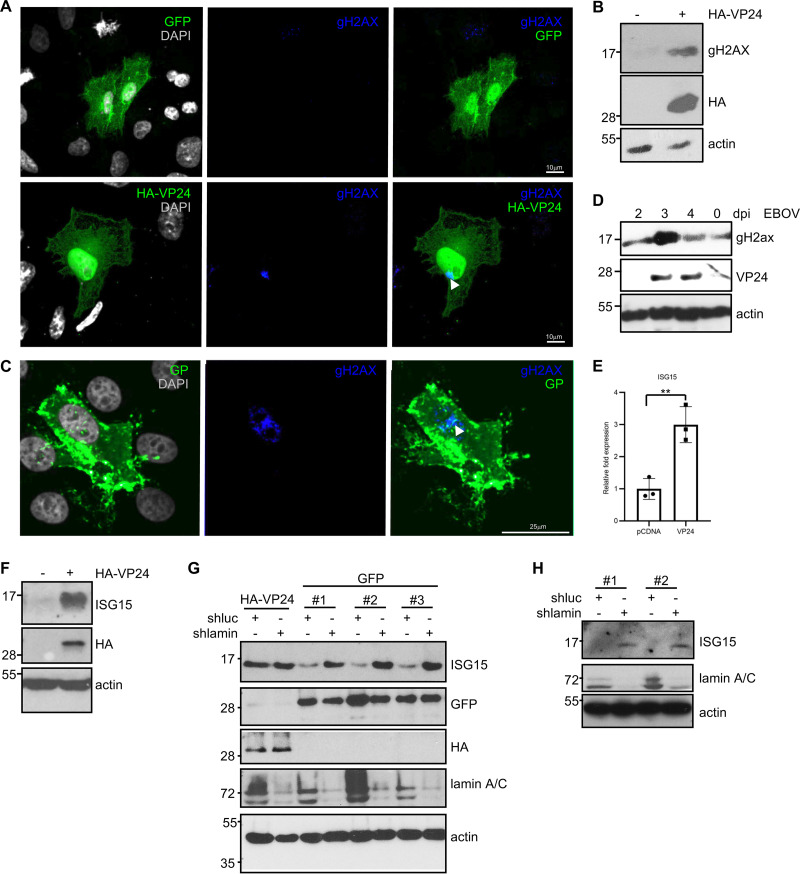
Induction of DNA damage and upregulation of ISG15 by VP24. (A) Immunofluorescence staining using anti-gH2AX antibody of Vero cells transfected with 0.3 μg of HA-VP24. Arrowhead indicates positive detection of gH2AX in cells expressing VP24. (B) Western blotting using anti-gH2AX antibody in Vero cells transfected with 0.3 μg of HA-VP24. (C) Immunofluorescence staining using anti-gH2AX antibody in uninfected cells (EBOV GP negative) and in cells infected with EBOV (EBOV GP positive). (D) Western blotting using anti-VP24 and anti-gH2AX antibodies in HeLa cells at different times after infection with EBOV. The 0 time corresponds to mock-infected cells that were in culture for 4 days. (E) Transcriptional transactivation of *ISG15* in response to VP24 expression by quantitative real-time PCR analysis. Columns are representative of the mean, and error bars represent the standard deviation of three biological replicates. Statistical significance was assessed by a Student’s *t* test. **, *P* < 0.01. (F) A549 cells transfected with 0.3 μg of pcDNA or HA-VP24 plasmids were analyzed by Western blotting with anti-ISG15 antibody. (G) HeLa cells stably transfected with shluc or shlamin A plasmids were transfected as indicated and analyzed by Western blotting with anti-ISG15 antibody. Three replicates of HeLa cells transfected with shluc or shlamin are shown. (H) A549 cells stably transfected with shluc or shlamin A plasmids were analyzed by Western blotting with anti-ISG15 antibody. Two replicates are shown.

Nuclear membrane disruption has been shown to correlate with DNA damage accumulation (56, 57). To assess levels of DNA damage, we analyzed the presence of foci of H2A histone family member X (gH2AX) in cells expressing VP24. We found foci of gH2AX in around 85% of VP24-expressing cells and in 13% of control cells (Fig. 9A). Western blotting also revealed an increase in gH2AX levels in those cells transfected with HA-VP24 (Fig. 9B), suggesting that expression of VP24 protein triggers DNA damage. Analysis of the presence of gH2AX foci in EBOV-infected cells was also analyzed. Both immunofluorescent gH2AX foci (Fig. 9C) and an increase in gH2AX levels were detected in those cells infected with EBOV (Fig. 9D).

DNA damage has been shown to induce ISG15 expression (57–60). Therefore, we decided to study the transcript and protein levels of ISG15 in cells expressing VP24. The transcript levels of ISG15 were significantly higher in cells transfected with VP24 than in pcDNA-transfected cells (Fig. 9E). In addition, Western blotting analysis of A549 or HeLa cells transfected with VP24 also revealed a significant increase in ISG15 protein levels (Fig. 9F and G, respectively), similar to that observed after downmodulation of lamin A/C (Fig. 9G and H).

### RanGAP and RanBP localization is not altered in VP24-expressing cells.

**FIG 10:**
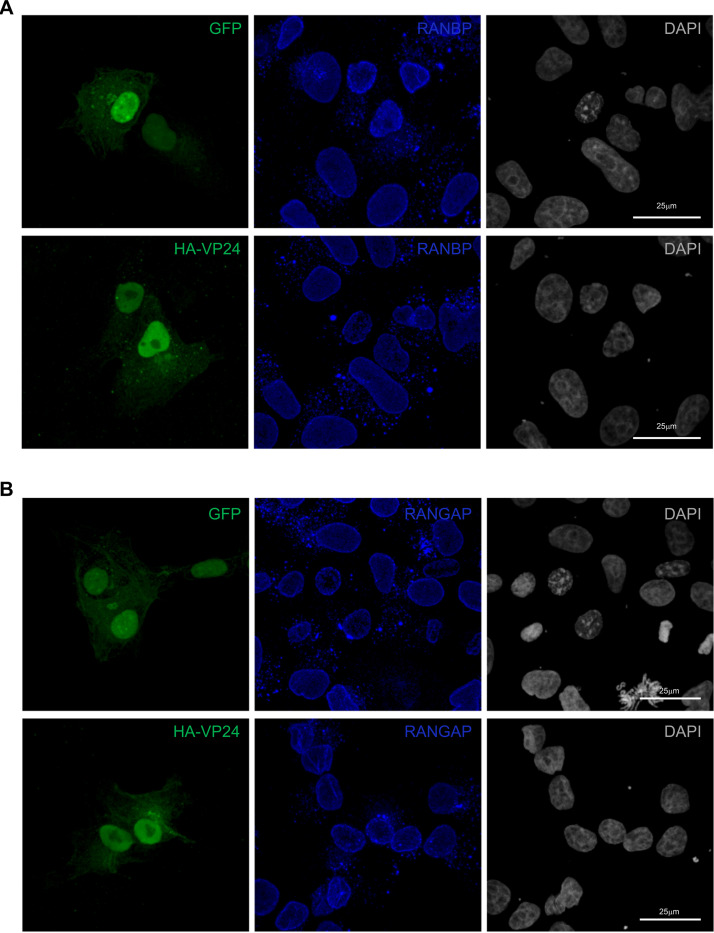
RanBP and RanGAP staining in VP24-expressing cells. Immunofluorescence staining using anti-RanBP (A) or anti-RanGAP (B) antibodies in Vero cells transfected with 0.3 μg GFP or HA-VP24. Chromosomes were stained with DAPI.

One of the functions of lamins is to anchor nuclear pores (61–63). VP24 disruption of lamins might then also trigger the mislocalization of proteins associated with the nuclear pore, such as Ran GTPase-activating protein (RanGAP) and Ran binding protein (RanBP). We analyzed Vero cells transfected with GFP or HA-VP24 using immunofluorescence staining with anti-RanGAP or anti-RanBP antibodies. Both RanGAP and RanBP were located at the nuclear envelope and in cytoplasmic aggregates independent of the expression of VP24 (Fig. 10A and B), suggesting that nuclear pore complexes are not altered by VP24.

### Translocation and downmodulation of BAF by VP24.

**FIG 11:**
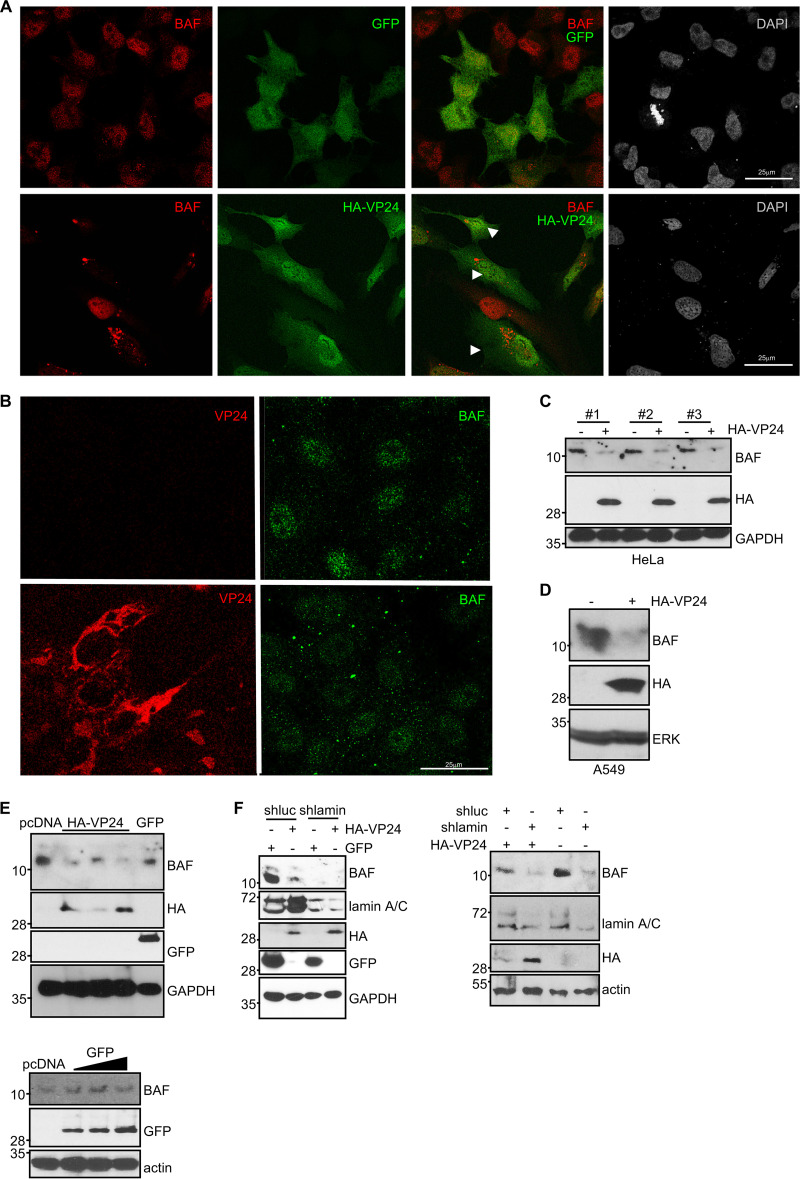
VP24 induces translocation and degradation of BAF. (A) Immunofluorescence staining using anti-BAF antibody in HeLa cells plated in 24-well plates and transfected with 0.3 μg of GFP or HA-VP24. Chromosomes were stained with DAPI. Arrowheads indicate cells expressing VP24. (B) Immunofluorescence staining using anti-BAF and anti-VP24 antibodies of cells infected with EBOV. (C) Western blotting of HeLa cells plated in 24-well plates and transfected with 0.3 μg of pcDNA or HA-VP24 using anti-BAF antibody. Three biological replicates are shown. (D) Western blotting of A549 cells plated in 24-well plates and transfected with 0.3 μg of pcDNA or HA-VP24 using anti-BAF antibody. (E) Western blotting of HeLa cells plated in 24-well plates and transfected with 0.3 μg of pcDNA or expressing different levels of HA-VP24 (top) or GFP (bottom) using anti-BAF antibody. (F) Western blotting of A549 cells stably transfected with shluc or shlamin plated in 24-well plates and transfected with 0.3 μg of GFP or HA-VP24 using anti-BAF antibody (left). Western blotting of HeLa cells stably transfected with shluc or shlamin plated in 24-well plates and transfected with 0.3 μg of GFP or HA-VP24 using anti-BAF antibody (right).

The interaction and structural interdependence of the nuclear envelope components lamin A, emerin, and BAF have been reported (24). Therefore, we decided to examine the distribution of BAF in cells expressing VP24. BAF was mainly detected inside the nucleus of 90% of untransfected or GFP-transfected HeLa cells, whereas BAF was mainly located in cytoplasmic granules rather than in the nucleus in 95% of those cells expressing VP24 (Fig. 11A). To evaluate whether BAF is also altered in response to EBOV infection, we carried out immunofluorescence staining using anti-BAF and anti-VP24 antibodies in HeLa cells infected with EBOV. BAF was mainly detected at the nucleus of the uninfected cells, whereas cytoplasmic dots were observed in those cells infected with EBOV (Fig. 11B). Together with the partners impact on BAF subcellular localization, phosphorylation has been also reported to influence BAF distribution (64). Therefore, we decided to analyze BAF protein in cells transfected with HA-VP24 by Western blotting. Interestingly, we also observed a clear decrease in the levels of BAF protein in HeLa (Fig. 11C) or A549 (Fig. 11D) cells transfected with VP24. We found an inverse correlation between VP24 and BAF protein levels (Fig. 11E). Furthermore, VP24 expression caused a decrease in BAF protein levels similar to that observed in cells with downmodulated lamin A/C protein (Fig. 11F). Reverse-transcription quantitative PCR (qRT-PCR) did not reveal any change in BAF expression between cells expressing or not expressing VP24 (data not shown), suggesting a posttranslational reduction of BAF.

**FIG 12:**
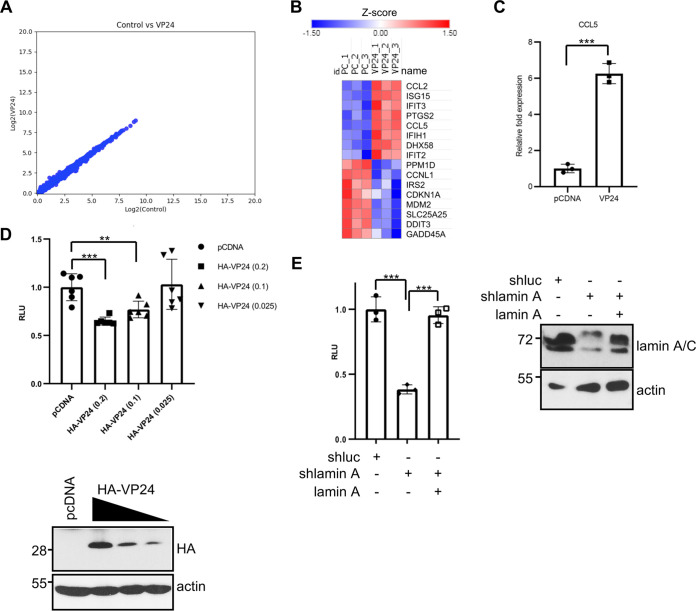
Transcriptional changes in response to VP24 expression. (A) Scatterplots of transcript expression data for Vero cells expressing VP24 obtained after RNA-seq analysis. (B) Heat map of genes transactivated or downmodulated (>1.3- to 1.4-fold) in response to HA-VP24 expression. (C) Transcriptional transactivation of *CCL5* in response to VP24 expression by quantitative real-time PCR analysis. Columns are representative of the mean, and error bars represent the standard deviation of three biological replicates. Statistical significance was assessed by a Student’s *t* test. ***, *P* < 0.001. (D) HeLa cells were cotransfected with the luciferase reporter pREP-CSF1-luciferase plasmid together with pcDNA-beta-gal and pcDNA or the indicated doses of HA-VP24 plasmids. At 36 h after transfection, luciferase production was analyzed. Columns are representative of the mean, and error bars represent the standard deviation of six biological replicates. Statistical significance was assessed by a Student’s *t* test (top). **, *P* < 0.01, ***, *P* < 0.001. Cell lysates from the experiment were analyzed by Western blotting for HA-VP24 expression (bottom). (E) HeLa cells stably transfected with shluc or shlamin were cotransfected with the luciferase reporter pREP-CSF1-luciferase plasmid together with pcDNA-beta-gal and pcDNA or a lamin A/C expression plasmid, as indicated. At 36 h after transfection, luciferase production was analyzed. Columns are representative of the mean, and error bars represent the standard deviation of three biological replicates. Similar results were obtained twice. Statistical significance was assessed by a Student’s *t* test (left). ***, *P* < 0.001. Cell lysates from the experiment were analyzed by Western blotting for lamin A/C expression (right).

BAF is a chromatin-binding protein, and it has a variety of functions, including the regulation of gene expression. It has been proposed that BAF limits basal inflammation, whereas ablation of BAF results in increased expression of some ISGs, including ISG15 (65, 66). ISG15 induction upon VP24 expression is then consistent with the observed BAF downmodulation. In order to identify additional host genes whose expression is altered by VP24 expression, we investigated the transcriptional pattern of cells expressing VP24 protein. Vero cells were cotransfected with VP24 or pcDNA together with GFP at a 10:1 ratio. At 24 h after transfection, fluorescence-activated cell sorter (FACS)-sorted GFP-expressing cells were subjected to RNA sequencing (RNA-seq) analysis. We did not observe genes with a >1.5-fold change (FC) in transcript levels in VP24-expressing cells (Fig. 12A). We only detected a small group of genes slightly, but significantly, upregulated or downmodulated (>1.3- to 1.4-fold) (Fig. 12B), indicating that VP24 expression alone provoked very little change in steady-state transcript abundance in Vero cells. Most of the downmodulated genes upon VP24 expression belong to the DNA damage response pathway, whereas the transactivated genes are related to the inflammatory response (Fig. 12B). Interestingly, one of the upregulated genes was *ISG15*, confirming our previous observations. In addition, to validate the differential expression results, qRT-PCR for another upregulated gene, *CCL5*, was performed. Results of the qRT-PCR assay confirmed the upregulation of the inflammatory cytokine *CCL5* in response to VP24 expression (Fig. 12C).

One of the genes activated by the BAF complex is the human colony-stimulating factor 1 (*CSF1*) gene (67). We then analyzed the transactivation of the CSF1-luciferase reporter in cells expressing VP24. HeLa cells were cotransfected with CSF1-luciferase together with beta-galactosidase and pcDNA or increasing doses of HA-VP24 plasmids, and 24 h after transfection, cells were harvested and assayed for luciferase and beta-galactosidase activity. We observed that VP24 inhibits luciferase expression in a dose-dependent manner (Fig. 12D). Similar inhibition of luciferase expression was observed after lamin downmodulation (Fig. 12E). Altogether, these results suggest that downregulation of BAF by VP24 reduces the expression of BAF-inducible genes.

## DISCUSSION

Here, we demonstrate that the EBOV VP24 protein interacts with emerin, lamin A/C, and lamin B, decreasing lamin A/C-emerin interaction and, consequently, inducing loss of nuclear membrane integrity. In agreement with a nuclear envelope disruption, cells expressing VP24 display several characteristics previously reported to be associated with a loss of integrity of the nuclear membrane, such as accumulation of DNA damage, alterations in nuclear size and shape, induction of ISG15, and MAPK pathway activation (53–60). Some viruses require nuclear proteins for replication; therefore, they have evolved mechanisms to overcome the barrier of the nuclear lamin (68). We propose that the lamin disruption by VP24 may facilitate access to some nuclear components or it may serve as a way to modulate signaling pathways. The MAPK pathway governs a wide range of cellular functions, including the regulation of innate immunity (69). Thus, ERK activation inhibits type I interferon production in different cell types (70–72). Consequently, many viruses usurp the pathway to their own benefit and enhance virus replication thanks to the induction of ERK phosphorylation (73–81). Induction of ERK phosphorylation may then be an additional mechanism by which VP24 interferes with IFN gene expression. But VP24 is not the unique EBOV protein able to activate ERK. Virus-like particles (VLPs) containing the viral matrix protein VP40 and the viral glycoprotein GP have also been reported to induce the activation of ERK1/2 (82), suggestive of the relevance of the MAPK pathway for EBOV replication.

Moreover, we show that expression of VP24 induces the translocation of BAF to the cytoplasm, its downmodulation, and repression of the BAF-dependent transactivation of CSF1, a regulator of the proliferation, differentiation, and survival of macrophages (83). VP24-mediated downmodulation of BAF may then be a mechanism by which VP24, cooperatively with EBOV VP35, suppresses maturation of infected human dendritic cells (11). BAF is also involved in the regulation of interferon-stimulated genes (ISGs). Upon nuclear envelope rupture, cGAS binds to the exposed genomic DNA and accumulates at the rupture site (51, 84), leading to proinflammatory activation, and its activity is prevented by BAF (65). Therefore, its downregulation as a consequence of VP24 expression may explain the counterintuitive transactivation of several proinflammatory genes also induced by IFN, such as ISG15. These results are in agreement with the VP24-mediated stimulation of the expression of cytokines, chemokines, and IFN detected at early times after EBOV infection (85). We then propose that, in addition to its well-known inhibitory activity of the IFN pathway, VP24 can still induce the transactivation of ISG15 and a subset of other ISGs in an IFN-independent manner, as previously reported (86, 87). Importantly, we also observed an alteration in BAF distribution in EBOV-infected cells. Overall, our data suggest that alterations in BAF localization and levels play a role in the inflammatory response activated by the virus.

RNA-seq experiments revealed that VP24 not only induces the upregulation of genes but also exerts transcriptional repression of several DNA damage response genes, such as *GADD45A*, *DDIT3*, or *CDKN1A*, reported previously as c-myc-regulated genes (88–90). Given the known association between ERK activation and the stabilization of c-myc mediated by its Ser62 phosphorylation (91), repression of these transcripts in response to VP24 expression may be mediated by ERK activation.

BAF has been previously shown to have antiviral activity, preventing vaccinia virus and herpes simplex type 1 (HSV-1) DNA replication (64), inhibiting retrovirus integration, and working as an epigenetic regulator of HSV lytic infection (92, 93). To counteract its antiviral effect, some viruses have evolved different strategies, such as inducing its phosphorylation (94) or altering its subcellular localization (64). To our knowledge, this study shows for the first time the downmodulation and mislocalization of BAF protein as a consequence of the expression of a viral protein, providing a likely mechanistic explanation for the displacement of BAF observed in Ebola virus-infected cells (Fig. 8B).

In summary, here we identify novel activities for EBOV VP24 protein with potential impact on EBOV replication to perturb the emerin-lamin interaction and to promote BAF downmodulation, leading to the activation of the MAPK pathway, DNA damage, and dysregulation of gene expression, hallmarks for most laminopathies (95). Even though BAF carries out essential functions for the cell, little is known about its regulation. Further studies will be necessary to identify the exact molecular mechanism by which BAF levels are modulated.

## MATERIALS AND METHODS

### Immunofluorescence.

Cells were fixed in 2% paraformaldehyde/phosphate-buffered saline (PBS) and incubated with 0.25% Triton X-100/PBS or digitonin in PBS to permeabilize the plasma and nuclear membrane or the plasma membrane, as indicated. Upon permeabilization, nonspecific binding sites were blocked with 2% bovine serum albumin (BSA)/PBS, and samples were incubated overnight with primary antibodies. Coverslips were extensively washed with PBS and further incubated with the appropriate Alexa-conjugated secondary antibodies for 1 h at room temperature. Nuclei were stained with 4′,6′-diamidino-2-phenylindole (DAPI), and preparations were mounted with ProLong Diamond antifade mounting medium (P36970) and visualized with a confocal Leica microscope.

### Biomolecular fluorescence complementation assay.

Cells grown on cover slides were transfected, and at 36 h, they were incubated for 3 h at 30°C. Then, cells were fixed and permeabilized with cold 100% methanol, blocked with 2% BSA/PBS, and incubated overnight with primary antibodies. Coverslips were washed extensively with PBS and further incubated with appropriate Alexa-conjugated secondary antibodies for 1 h at room temperature. Nuclei were stained with DAPI, and preparations were mounted with ProLong Diamond antifade mounting medium (P36970) and visualized with a confocal Leica microscope.

### Cells, plasmids, and reagents.

HEK-293, HeLa, Vero, HUH-7, and A549 cells were cultured in Dulbecco’s modified Eagle’s medium (DMEM) supplemented with 10% fetal bovine serum (FBS), 1% l-glutamine, and 1% penicillin/streptomycin. Transfection experiments were performed using polyethylenimine (PEI), according to the manufacturer’s instructions. The plasmid encoding HA-tagged Mayinga EBOV VP24 (HA-VP24) has been previously reported (96). The plasmid encoding GFP-VP24 was generated by subcloning of the cDNA coding VP24 into the pEGFP-C1 vector (Clontech). The plasmids pTRIP-SFFV-EGFP-NLS and pTRIP-CMV-GFP-Flag-cGAS were kindly provided by Nicolas Manel (Addgene plasmids 86677 and 86675, respectively) (97). pLKO-sh-lamin A and PLKO-sh-luciferase were previously described (98). The plasmids YN-VP24, YC-laminA, YC-emerin, and YN-emerin were obtained by cloning of the coding region in the plasmids pCAGGS-eYN and pCAGGS-eYC (99) with the oligonucleotides listed in Table 1. PREP4-CSF1-luciferase plasmid was kindly provided by Keji Zhao (67). The EBOV minigenome luciferase reporter and the pcAGGS expression plasmids for Zaire EBOV strain Mayinga L, VP30, VP35, and NP (38) were kind gifts from Christopher Basler. Plasmids of the Zaire EBOV Makona minigenome system, VP35, NP, VP30, and L, were previously reported (100). Plasmids used in this study, unless specified otherwise, are derived from the Mayinga strain.

**TABLE 1:** Oligonucleotides for cloning

Oligonucleotide	Sequence
Xho-link-VP24-F	5′-GGCCCTCGAGCTCAAGCTTCGAATTCTATGGCTAAAGCTACGGGACGATAC-3′
NheI-VP24-R	5′-GCTAGCTCAGATAGCAAGAGAGCTA-3′
Xho-link-emerin-F	5′-GGCCCTCGAGCTCAAGCTTCGAATTCTATGGACAACTACGCAGATCTTTCGG-3′
NheI-emerin-R	5′-GCTAGCTCAGAAGGGGTTGCCTTCTTC-3′
Xho-link-laminA-F	5′-GGCCCTCGAGCTCAAGCTTCGAATTCTATGGAGACCCCGTCCCAGCGGCGCGC-3′
NheI-laminA-R	5′-GCTAGCTCACATGATGCTGCAGTTCTGGGG-3′

### Quantitative PCR.

Total RNA was purified with the RNeasy minikit (Qiagen), and reverse transcription (RT-PCR) was performed using the reverse transcription system kit (Promega). qRT-PCR was performed using SYBR green power PCR master mix in a RealPlex 4 thermocycler (Eppendorf). The oligonucleotides used are listed in Table 2.

**TABLE 2:** Oligonucleotides for RT-PCR

Oligonucleotide	Sequence
GAPDH-Vero-qRT-F	5′-GTGAAGGTCGGAGTCAACGG-3′
GAPDH-Vero-qRT-R	5′-AAGACGCCAGTGGACTCCA-3′
ISG15-Vero-qRT-F	5′-GAGAGGCAGCGAACTCATCT-3′
ISG15-Vero-qRT-R	5′-CTTCAGCTCTGACACCGACA-3′
CCL5-Vero-qRT-F	5′-GCTGTCATCCTCATTGCTACAG-3′
CCL5-Vero-qRT-R	5′-TGGTGTAGAAATACTCCTTGATGTG-3′
EBOV L-qRT-F	5′-CCATCTACATCGGTGGAGCC-3′
EBOV L-qRT-R	5′-GTGGTCGTTGATGGTGGTCT-3′
EBOV VP30-qRT-F	5′-CTCGCCAAAGGAATGCAAGG-3′
EBOV VP30-qRT-R	5′-AAAGGGTCGCTACAGACGTT-3′

### Reporter assay.

HeLa cells were cotransfected with PREP4-CSF1-luciferase plasmid, the pcDNA-beta-galactosidase plasmid, and the indicated plasmids. At 36 h after transfection, cells were harvested and analyzed. An interferon reporter assay was done as reported previously (96). Firefly luciferase values were normalized to beta-galactosidase values. Fold induction for each sample was then determined relative to the normalized luciferase activity value for pcDNA-transfected cells. Statistical significance was assessed using a Student’s *t* test.

### Western blotting analysis and antibodies.

For Western blotting, cells were washed in PBS, scraped in SDS gel-loading buffer, and boiled for 5 min. Proteins of total extracts were separated by SDS-PAGE and transferred to nitrocellulose membranes. Monoclonal antibody against HA was purchased from BioLegend. Anti-HA goat was from Bethyl Laboratories. Anti-emerin and anti-lamin A/C antibodies were from Abcam and Cell Signaling. Anti-tubulin, anti-gH2AX, and anti-ERK were from Cell Signaling. Anti-GAPDH, anti-actin, anti-lamin B, anti-RanGAP, anti-RanBP, and anti-phospho-ERK antibodies were from Santa Cruz Biotechnology. Anti-VP24 was from Biorbyt. Anti-VP35 and anti-NP were from Genetex. Anti-BAF antibody was from Abcam.

### Infection.

HeLa cells at 90% confluence were infected with EBOV (NC_002549.1) at a multiplicity of infection (MOI) of 1. All infection experiments were carried out by experienced personnel wearing positive pressure protection suits at the biosafety level 4 (BSL4) laboratory of the Bernhard Nocht Institute for Tropical Medicine in Hamburg.

### Immunoprecipitation assay.

Cells were lysed in radioimmunoprecipitation assay (RIPA) or BC-100 buffer at 4°C, centrifuged at 15,800 × *g* for 10 min, and immunoprecipitated overnight at 4°C after addition of the specified antibody and 30 μl of 50% protein G-sepharose (Life Technologies). Beads were then washed four times with lysis buffer and resuspended in 30 μl of loading buffer.

### Minigenome assay.

This assay was performed as previously described (38). Briefly, 1 × 10^6^ HEK-293 cells were cotransfected with plasmids for the expression of the T7 polymerase (0.4 μg), pCAGGS-NP (0.49 μg), pCAGGS-VP30 (0.2 μg), pCAGGS-VP35 (0.25), pCAGGS-L (1 μg), and GFP or pTM1-eMGLuc (0.5 mg) of the EBOV Mayinga minigenome (38) together with pcDNA or HA-VP24. At 48 h after transfection, luciferase activity was analyzed.

### RNA-seq.

RNA-seq was carried out at the CNIC Genomics Unit. RNA quantity was measured using a Nanodrop (Thermo Scientific), and RNA integrity was measured with an Agilent 6000 Pico kit and Bioanalyzer. Total RNA (200 ng) was used to generate barcoded RNA-seq libraries using the NEBNext Ultra RNA library preparation kit (New England Biolabs). Briefly, poly A+ RNA was purified using poly T oligo-attached magnetic beads followed by fragmentation and then first and second cDNA strand synthesis. Next, cDNA 3′ ends were adenylated, and the adapters were ligated followed by PCR library amplification. Finally, the size of the libraries was checked using the Agilent 2100 Bioanalyzer DNA 1000 chip, and library concentration was determined using the Qubit fluorometer (Life Technologies). Libraries were sequenced on a HiSeq2500 (Illumina) to generate 60 bases single reads and processed with RTA v1.18.66.3. FastQ files for each sample were obtained using bcl2fastq v2.20.0.422 software (Illumina). Sequencing reads were trimmed of Illumina adapters using cutadapt 1.16 and then aligned to the African green monkey reference transcriptome (ChlSab1 v92) and quantified with RSem v1.3.1 (101, 102). Raw counts were normalized with transcripts per million (TPM) and trimmed mean of M values (TMM) methods, transformed into log_2_ expression (log_2_ [rawCount + 1]), and compared to calculate fold change (FC) and corrected *P* values. Two groups were too similar, so we could not detect any significant differential expressed with the limits of a log_2_ FC of >1 (2×) and a corrected *P* value of <0.05. We reduced the limits to a log_2_ FC of >0.4 (1.32×) and a *P* value of <0.05 and tested some of the candidates by qRT-PCR. Only mRNAs detected in almost 3 samples were used in the analysis. Heat maps were created with the Morpheus web app from the Broad Institute.

### Image processing.

Confocal images were analyzed for nuclear area and circularity (= 4 π [area/perimeter^2^]) using ImageJ software.

### Data availability.

The data sets generated during the current study are available in the following link https://www.ncbi.nlm.nih.gov/geo/query/acc.cgi?acc=GSE155936.

## References

[1] Noda T, Halfmann P, Sagara H, Kawaoka Y. 2007. Regions in Ebola virus VP24 that are important for nucleocapsid formation. J Infect Dis 196:S247–S250. doi:10.1086/520596. 17940956

[2] Han Z, Boshra H, Sunyer JO, Zwiers SH, Paragas J, Harty RN. 2003. Biochemical and functional characterization of the Ebola virus VP24 protein: implications for a role in virus assembly and budding. J Virol 77:1793–1800. doi:10.1128/JVI.77.3.1793-1800.2003. 12525613 PMC140957

[3] Huang Y, Xu L, Sun Y, Nabel GJ. 2002. The assembly of Ebola virus nucleocapsid requires virion-associated proteins 35 and 24 and posttranslational modification of nucleoprotein. Mol Cell 10:307–316. doi:10.1016/S1097-2765(02)00588-9. 12191476

[4] Elliott LH, Kiley MP, McCormick JB. 1985. Descriptive analysis of Ebola virus proteins. Virology 147:169–176. doi:10.1016/0042-6822(85)90236-3. 4060597

[5] Reid SP, Valmas C, Martinez O, Sanchez FM, Basler CF. 2007. Ebola virus VP24 proteins inhibit the interaction of NPI-1 subfamily karyopherin alpha proteins with activated STAT1. J Virol 81:13469–13477. doi:10.1128/JVI.01097-07. 17928350 PMC2168840

[6] Reid SP, Leung LW, Hartman AL, Martinez O, Shaw ML, Carbonnelle C, Volchkov VE, Nichol ST, Basler CF. 2006. Ebola virus VP24 binds karyopherin alpha1 and blocks STAT1 nuclear accumulation. J Virol 80:5156–5167. doi:10.1128/JVI.02349-05. 16698996 PMC1472181

[7] Mateo M, Reid SP, Leung LW, Basler CF, Volchkov VE. 2010. Ebolavirus VP24 binding to karyopherins is required for inhibition of interferon signaling. J Virol 84:1169–1175. doi:10.1128/JVI.01372-09. 19889762 PMC2798383

[8] Guito JC, Albariño CG, Chakrabarti AK, Towner JS. 2017. Novel activities by ebolavirus and marburgvirus interferon antagonists revealed using a standardized in vitro reporter system. Virology 501:147–165. doi:10.1016/j.virol.2016.11.015. 27930961 PMC11524407

[9] Zhang AP, Bornholdt ZA, Liu T, Abelson DM, Lee DE, Li S, Woods VL, Saphire EO. 2012. The Ebola virus interferon antagonist VP24 directly binds STAT1 and has a novel, pyramidal fold. PLoS Pathog 8:e1002550. doi:10.1371/journal.ppat.1002550. 22383882 PMC3285596

[10] Shabman RS, Gulcicek EE, Stone KL, Basler CF. 2011. The Ebola virus VP24 protein prevents hnRNP C1/C2 binding to karyopherin α1 and partially alters its nuclear import. J Infect Dis 204:S904–S910. doi:10.1093/infdis/jir323. 21987768 PMC3189985

[11] Lubaki NM, Ilinykh P, Pietzsch C, Tigabu B, Freiberg AN, Koup RA, Bukreyev A. 2013. The lack of maturation of Ebola virus-infected dendritic cells results from the cooperative effect of at least two viral domains. J Virol 87:7471–7485. doi:10.1128/JVI.03316-12. 23616668 PMC3700277

[12] Mateo M, Carbonnelle C, Martinez MJ, Reynard O, Page A, Volchkova VA, Volchkov VE. 2011. Knockdown of Ebola virus VP24 impairs viral nucleocapsid assembly and prevents virus replication. J Infect Dis 204:S892–S896. doi:10.1093/infdis/jir311. 21987766

[13] García-Dorival I, Wu W, Dowall S, Armstrong S, Touzelet O, Wastling J, Barr JN, Matthews D, Carroll M, Hewson R, Hiscox JA. 2014. Elucidation of the Ebola virus VP24 cellular interactome and disruption of virus biology through targeted inhibition of host-cell protein function. J Proteome Res 13:5120–5135. doi:10.1021/pr500556d. 25158218

[14] Batra J, Hultquist JF, Liu D, Shtanko O, Von Dollen J, Satkamp L, Jang GM, Luthra P, Schwarz TM, Small GI, Arnett E, Anantpadma M, Reyes A, Leung DW, Kaake R, Haas P, Schmidt CB, Schlesinger LS, LaCount DJ, Davey RA, Amarasinghe GK, Basler CF, Krogan NJ. 2018. Protein interaction mapping identifies RBBP6 as a negative regulator of Ebola virus replication. Cell 175:1917–1930. doi:10.1016/j.cell.2018.08.044. 30550789 PMC6366944

[15] Lin F, Blake DL, Callebaut I, Skerjanc IS, Holmer L, McBurney MW, Paulin-Levasseur M, Worman HJ. 2000. MAN1, an inner nuclear membrane protein that shares the LEM domain with lamina-associated polypeptide 2 and emerin. J Biol Chem 275:4840–4847. doi:10.1074/jbc.275.7.4840. 10671519

[16] Paddy MR, Belmont AS, Saumweber H, Agard DA, Sedat JW. 1990. Interphase nuclear envelope lamins form a discontinuous network that interacts with only a fraction of the chromatin in the nuclear periphery. Cell 62:89–106. doi:10.1016/0092-8674(90)90243-8. 2194675

[17] Solovei I, Wang AS, Thanisch K, Schmidt CS, Krebs S, Zwerger M, Cohen TV, Devys D, Foisner R, Peichl L, Herrmann H, Blum H, Engelkamp D, Stewart CL, Leonhardt H, Joffe B. 2013. LBR and lamin A/C sequentially tether peripheral heterochromatin and inversely regulate differentiation. Cell 152:584–598. doi:10.1016/j.cell.2013.01.009. 23374351

[18] Lloyd DJ, Trembath RC, Shackleton S. 2002. A novel interaction between lamin A and SREBP1: implications for partial lipodystrophy and other laminopathies. Hum Mol Genet 11:769–777. doi:10.1093/hmg/11.7.769. 11929849

[19] Margalit A, Vlcek S, Gruenbaum Y, Foisner R. 2005. Breaking and making of the nuclear envelope. J Cell Biochem 95:454–465. doi:10.1002/jcb.20433. 15832341

[20] Rodríguez S, Eriksson M. 2010. Evidence for the involvement of lamins in aging. Curr Aging Sci 3:81–89. doi:10.2174/1874609811003020081. 20044904

[21] Wilson KL, Foisner R. 2010. Lamin-binding proteins. Cold Spring Harb Perspect Biol 2:a000554. doi:10.1101/cshperspect.a000554. 20452940 PMC2845209

[22] Gruenbaum Y, Margalit A, Goldman RD, Shumaker DK, Wilson KL. 2005. The nuclear lamina comes of age. Nat Rev Mol Cell Biol 6:21–31. doi:10.1038/nrm1550. 15688064

[23] Dechat T, Adam SA, Goldman RD. 2009. Nuclear lamins and chromatin: when structure meets function. Adv Enzyme Regul 49:157–166. doi:10.1016/j.advenzreg.2008.12.003. 19154754 PMC3253622

[24] Qi R, Xu N, Wang G, Ren H, Li S, Lei J, Lin Q, Wang L, Gu X, Zhang H, Jiang Q, Zhang C. 2015. The lamin-A/C-LAP2α-BAF1 protein complex regulates mitotic spindle assembly and positioning. J Cell Sci 128:2830–2841. doi:10.1242/jcs.164566. 26092935

[25] Liu J, Lee KK, Segura-Totten M, Neufeld E, Wilson KL, Gruenbaum Y. 2003. MAN1 and emerin have overlapping function(s) essential for chromosome segregation and cell division in *Caenorhabditis elegans*. Proc Natl Acad Sci USA 100:4598–4603. doi:10.1073/pnas.0730821100. 12684533 PMC153601

[26] Samson C, Petitalot A, Celli F, Herrada I, Ropars V, Le Du MH, Nhiri N, Jacquet E, Arteni AA, Buendia B, Zinn-Justin S. 2018. Structural analysis of the ternary complex between lamin A/C, BAF and emerin identifies an interface disrupted in autosomal recessive progeroid diseases. Nucleic Acids Res 46:10460–10473. doi:10.1093/nar/gky736. 30137533 PMC6212729

[27] Hennig T, O’Hare P. 2015. Viruses and the nuclear envelope. Curr Opin Cell Biol 34:113–121. doi:10.1016/j.ceb.2015.06.002. 26121672

[28] Herrada I, Samson C, Velours C, Renault L, Östlund C, Chervy P, Puchkov D, Worman HJ, Buendia B, Zinn-Justin S. 2015. Muscular dystrophy mutations impair the nuclear envelope emerin self-assembly properties. ACS Chem Biol 10:2733–2742. doi:10.1021/acschembio.5b00648. 26415001 PMC4869717

[29] Berk JM, Simon DN, Jenkins-Houk CR, Westerbeck JW, Grønning-Wang LM, Carlson CR, Wilson KL. 2014. The molecular basis of emerin-emerin and emerin-BAF interactions. J Cell Sci 127:3956–3969. 25052089 10.1242/jcs.148247 PMC4163644

[30] He F, Melén K, Maljanen S, Lundberg R, Jiang M, Österlund P, Kakkola L, Julkunen I. 2017. Ebolavirus protein VP24 interferes with innate immune responses by inhibiting interferon-λ1 gene expression. Virology 509:23–34. doi:10.1016/j.virol.2017.06.002. 28595092

[31] Sullivan T, Escalante-Alcalde D, Bhatt H, Anver M, Bhat N, Nagashima K, Stewart CL, Burke B. 1999. Loss of A-type lamin expression compromises nuclear envelope integrity leading to muscular dystrophy. J Cell Biol 147:913–920. doi:10.1083/jcb.147.5.913. 10579712 PMC2169344

[32] Fairley EA, Kendrick-Jones J, Ellis JA. 1999. The Emery-Dreifuss muscular dystrophy phenotype arises from aberrant targeting and binding of emerin at the inner nuclear membrane. J Cell Sci 112:2571–2582. doi:10.1242/jcs.112.15.2571. 10393813

[33] Tsuchiya Y, Hase A, Ogawa M, Yorifuji H, Arahata K. 2001. Distinct regions specify the nuclear membrane targeting of emerin, the responsible protein for Emery-Dreifuss muscular dystrophy. Eur J Biochem 259:859–865. doi:10.1046/j.1432-1327.1999.00112.x. 10092874

[34] Clements L, Manilal S, Love DR, Morris GE. 2000. Direct interaction between emerin and lamin A. Biochem Biophys Res Commun 267:709–714. doi:10.1006/bbrc.1999.2023. 10673356

[35] Vaughan A, Alvarez-Reyes M, Bridger JM, Broers JL, Ramaekers FC, Wehnert M, Morris GE, Whitfield WGF, Hutchison CJ. 2001. Both emerin and lamin C depend on lamin A for localization at the nuclear envelope. J Cell Sci 114:2577–2590. doi:10.1242/jcs.114.14.2577. 11683386

[36] Kolb T, Maaß K, Hergt M, Aebi U, Herrmann H. 2011. Lamin A and lamin C form homodimers and coexist in higher complex forms both in the nucleoplasmic fraction and in the lamina of cultured human cells. Nucleus 2:425–433. doi:10.4161/nucl.2.5.17765. 22033280

[37] Watanabe S, Noda T, Halfmann P, Jasenosky L, Kawaoka Y. 2007. Ebola virus (EBOV) VP24 inhibits transcription and replication of the EBOV genome. J Infect Dis 196:S284–S290. doi:10.1086/520582. 17940962

[38] Edwards MR, Pietzsch C, Vausselin T, Shaw ML, Bukreyev A, Basler CF. 2015. High-throughput minigenome system for identifying small-molecule inhibitors of Ebola virus replication. ACS Infect Dis 1:380–387. doi:10.1021/acsinfecdis.5b00053. 26284260 PMC4537067

[39] Hoenen T, Jung S, Herwig A, Groseth A, Becker S. 2010. Both matrix proteins of Ebola virus contribute to the regulation of viral genome replication and transcription. Virology 403:56–66. doi:10.1016/j.virol.2010.04.002. 20444481

[40] Nanbo A, Watanabe S, Halfmann P, Kawaoka Y. 2013. The spatio-temporal distribution dynamics of Ebola virus proteins and RNA in infected cells. Sci Rep 3:1206. doi:10.1038/srep01206. 23383374 PMC3563031

[41] Watt A, Moukambi F, Banadyga L, Groseth A, Callison J, Herwig A, Ebihara H, Feldmann H, Hoenen T. 2014. A novel life cycle modeling system for Ebola virus shows a genome length-dependent role of VP24 in virus infectivity. J Virol 88:10511–10524. doi:10.1128/JVI.01272-14. 24965473 PMC4178905

[42] Banadyga L, Hoenen T, Ambroggio X, Dunham E, Groseth A, Ebihara H. 2017. Ebola virus VP24 interacts with NP to facilitate nucleocapsid assembly and genome packaging. Sci Rep 7:7698. doi:10.1038/s41598-017-08167-8. 28794491 PMC5550494

[43] Weis K. 2003. Regulating access to the genome: nucleocytoplasmic transport throughout the cell cycle. Cell 112:441–451. doi:10.1016/S0092-8674(03)00082-5. 12600309

[44] Raharjo WH, Enarson P, Sullivan T, Stewart CL, Burke B. 2001. Nuclear envelope defects associated with LMNA mutations cause dilated cardiomyopathy and Emery-Dreifuss muscular dystrophy. J Cell Sci 114:4447–4457. doi:10.1242/jcs.114.24.4447. 11792810

[45] Östlund C, Bonne G, Schwartz K, Worman HJ. 2001. Properties of lamin A mutants found in Emery-Dreifuss muscular dystrophy, cardiomyopathy and Dunnigan-type partial lipodystrophy. J Cell Sci 114:4435–4445. doi:10.1242/jcs.114.24.4435. 11792809

[46] Holt I, Östlund C, Stewart CL, Man Nt, Worman HJ, Morris GE. 2003. Effect of pathogenic mis-sense mutations in lamin A on its interaction with emerin in vivo. J Cell Sci 116:3027–3035. doi:10.1242/jcs.00599. 12783988

[47] Hatch EM, Fischer AH, Deerinck TJ, Hetzer MW. 2013. Catastrophic nuclear envelope collapse in cancer cell micronuclei. Cell 154:47–60. doi:10.1016/j.cell.2013.06.007. 23827674 PMC3749778

[48] Adam SA, Marr RS, Gerace L. 1990. Nuclear protein import in permeabilized mammalian cells requires soluble cytoplasmic factors. J Cell Biol 111:807–816. doi:10.1083/jcb.111.3.807. 2391365 PMC2116268

[49] Plutner H, Davidson HW, Saraste J, Balch WE. 1992. Morphological analysis of protein transport from the ER to Golgi membranes in digitonin-permeabilized cells: role of the P58 containing compartment. J Cell Biol 119:1097–1116. doi:10.1083/jcb.119.5.1097. 1447290 PMC2289727

[50] Barrowman J, Hamblet C, George CM, Michaelis S. 2008. Analysis of prelamin A biogenesis reveals the nucleus to be a CaaX processing compartment. Mol Biol Cell 19:5398–5408. doi:10.1091/mbc.e08-07-0704. 18923140 PMC2592638

[51] Denais CM, Gilbert RM, Isermann P, McGregor AL, te Lindert M, Weigelin B, Davidson PM, Friedl P, Wolf K, Lammerding J. 2016. Nuclear envelope rupture and repair during cancer cell migration. Science 352:353–358. doi:10.1126/science.aad7297. 27013428 PMC4833568

[52] Nikolova V, Leimena C, McMahon AC, Tan JC, Chandar S, Jogia D, Kesteven SH, Michalicek J, Otway R, Verheyen F, Rainer S, Stewart CL, Martin D, Feneley MP, Fatkin D. 2004. Defects in nuclear structure and function promote dilated cardiomyopathy in lamin A/C-deficient mice. J Clin Invest 113:357–369. doi:10.1172/JCI200419448. 14755333 PMC324538

[53] Muchir A, Shan J, Bonne G, Lehnart SE, Worman HJ. 2008. Inhibition of extracellular signal-regulated kinase signaling to prevent cardiomyopathy caused by mutation in the gene encoding A-type lamins. Hum Mol Genet 18:241–247. doi:10.1093/hmg/ddn343. 18927124 PMC2638780

[54] Muchir A, Worman HJ. 2007. Emery-Dreifuss muscular dystrophy. Curr Neurol Neurosci Rep 7:78–83. doi:10.1007/s11910-007-0025-3. 17217858

[55] Muchir A, Pavlidis P, Bonne G, Hayashi YK, Worman HJ. 2007. Activation of MAPK in hearts of EMD null mice: similarities between mouse models of X-linked and autosomal dominant Emery Dreifuss muscular dystrophy. Hum Mol Genet 16:1884–1895. doi:10.1093/hmg/ddm137. 17567779

[56] Chen NY, Kim P, Weston TA, Edillo L, Tu Y, Fong LG, Young SG. 2018. Fibroblasts lacking nuclear lamins do not have nuclear blebs or protrusions but nevertheless have frequent nuclear membrane ruptures. Proc Natl Acad Sci USA 115:10100–10105. doi:10.1073/pnas.1812622115. 30224463 PMC6176609

[57] Kreienkamp R, Graziano S, Coll-Bonfill N, Bedia-Diaz G, Cybulla E, Vindigni A, Dorsett D, Kubben N, Batista LFZ, Gonzalo S. 2018. A cell-intrinsic interferon-like response links replication stress to cellular aging caused by progerin. Cell Rep 22:2006–2015. doi:10.1016/j.celrep.2018.01.090. 29466729 PMC5848491

[58] Gentile M, Latonen L, Laiho M. 2003. Cell cycle arrest and apoptosis provoked by UV radiation-induced DNA damage are transcriptionally highly divergent responses. Nucleic Acids Res 31:4779–4790. doi:10.1093/nar/gkg675. 12907719 PMC169943

[59] Liu M, Hummer BT, Li X, Hassel BA. 2004. Camptothecin induces the ubiquitin-like protein, ISG15, and enhances ISG15 conjugation in response to interferon. J Interferon Cytokine Res 24:647–654. doi:10.1089/jir.2004.24.647. 15684817

[60] Park JH, Yang SW, Park JM, Ka SH, Kim JH, Kong YY, Jeon YJ, Seol JH, Chung CH. 2016. Positive feedback regulation of p53 transactivity by DNA damage-induced ISG15 modification. Nat Commun 7:12513. doi:10.1038/ncomms12513. 27545325 PMC4996943

[61] Smythe C, Jenkins HE, Hutchison CJ. 2000. Incorporation of the nuclear pore basket protein nup153 into nuclear pore structures is dependent upon lamina assembly: evidence from cell-free extracts of *Xenopus* eggs. EMBO J 19:3918–3931. doi:10.1093/emboj/19.15.3918. 10921874 PMC306609

[62] Al-Haboubi T, Shumaker DK, Köser J, Wehnert M, Fahrenkrog B. 2011. Distinct association of the nuclear pore protein Nup153 with A- and B-type lamins. Nucleus 2:500–509. doi:10.4161/nucl.2.5.17913. 21983083

[63] Lussi YC, Hügi I, Laurell E, Kutay U, Fahrenkrog B. 2011. The nucleoporin Nup88 is interacting with nuclear lamin A. Mol Biol Cell 22:1080–1090. doi:10.1091/mbc.e10-05-0463. 21289091 PMC3069011

[64] Jamin A, Thunuguntla P, Wicklund A, Jones C, Wiebe MS. 2014. Barrier to auto integration factor becomes dephosphorylated during HSV-1 infection and can act as a host defense by impairing viral DNA replication and gene expression. PLoS One 9:e100511. doi:10.1371/journal.pone.0100511. 24945635 PMC4063967

[65] Guey B, Wischnewski M, Decout A, Makasheva K, Kaynak M, Sakar MS, Fierz B, Ablasser A. 2020. BAF restricts cGAS on nuclear DNA to prevent innate immune activation. Science 369:823–828. doi:10.1126/science.aaw6421. 32792394

[66] Ma H, Qian W, Bambouskova M, Collins PL, Porter SI, Byrum AK, Zhang R, Artyomov M, Oltz EM, Mosammaparast N, Miner JJ, Diamond MS. 2020. Barrier-to-autointegration factor 1 protects against a basal cGAS-STING response. mBio 11:e00136-20. doi:10.1128/mBio.00136-20. 32156810 PMC7064753

[67] Liu R, Liu H, Chen X, Kirby M, Brown PO, Zhao K. 2001. Regulation of CSF1 promoter by the SWI/SNF-like BAF complex. Cell 106:309–318. doi:10.1016/S0092-8674(01)00446-9. 11509180

[68] Walker EJ, Ghildyal R. 2017. Editorial: viral interactions with the nucleus. Front Microbiol 8:951. doi:10.3389/fmicb.2017.00951. 28603520 PMC5445102

[69] Dong C, Davis RJ, Flavell RA. 2002. MAP kinases in the immune response. Annu Rev Immunol 20:55–72. doi:10.1146/annurev.immunol.20.091301.131133. 11861597

[70] Janovec V, Aouar B, Font-Haro A, Hofman T, Trejbalova K, Weber J, Chaperot L, Plumas J, Olive D, Dubreuil P, Nunès JA, Stranska R, Hirsch I. 2018. The MEK1/2-ERK pathway inhibits type I IFN production in plasmacytoid dendritic cells. Front Immunol 9:364. doi:10.3389/fimmu.2018.00364. 29535732 PMC5835309

[71] Christian SL, Zu D, Licursi M, Komatsu Y, Pongnopparat T, Codner DA, Hirasawa K. 2012. Suppression of IFN-induced transcription underlies IFN defects generated by activated Ras/MEK in human cancer cells. PLoS One 7:e44267. doi:10.1371/journal.pone.0044267. 22970192 PMC3436881

[72] Chen Y, Chen J, Wang H, Shi J, Wu K, Liu S, Liu Y, Wu J. 2013. HCV-induced miR-21 contributes to evasion of host immune system by targeting MyD88 and IRAK1. PLoS Pathog 9:e1003248. doi:10.1371/journal.ppat.1003248. 23633945 PMC3635988

[73] Zhao LJ, Wang L, Ren H, Cao J, Li L, Ke JS, Qi ZT. 2005. Hepatitis C virus E2 protein promotes human hepatoma cell proliferation through the MAPK/ERK signaling pathway via cellular receptors. Exp Cell Res 305:23–32. doi:10.1016/j.yexcr.2004.12.024. 15777784

[74] Schümann M, Dobbelstein M. 2006. Adenovirus-induced extracellular signal-regulated kinase phosphorylation during the late phase of infection enhances viral protein levels and virus progeny. Cancer Res 66:1282–1288. doi:10.1158/0008-5472.CAN-05-1484. 16452180

[75] Liao B, Zhou H, Liang H, Li C. 2017. Regulation of ERK and AKT pathways by hepatitis B virus X protein via the Notch1 pathway in hepatocellular carcinoma. Int J Oncol 51:1449–1459. doi:10.3892/ijo.2017.4126. 29048612 PMC5643068

[76] Woodson EN, Kedes DH. 2012. Distinct roles for extracellular signal-regulated kinase 1 (ERK1) and ERK2 in the structure and production of a primate gammaherpesvirus. J Virol 86:9721–9736. doi:10.1128/JVI.00695-12. 22740395 PMC3446570

[77] Colao I, Pennisi R, Venuti A, Nygårdas M, Heikkilä O, Hukkanen V, Sciortino MT. 2017. The ERK-1 function is required for HSV-1-mediated G1/S progression in HEP-2 cells and contributes to virus growth. Sci Rep 7:9176. doi:10.1038/s41598-017-09529-y. 28835716 PMC5569015

[78] Rodems SM, Spector DH. 1998. Extracellular signal-regulated kinase activity is sustained early during human cytomegalovirus infection. J Virol 72:9173–9180. doi:10.1128/JVI.72.11.9173-9180.1998. 9765464 PMC110336

[79] Sharma-Walia N, Krishnan HH, Naranatt PP, Zeng L, Smith MS, Chandran B. 2005. ERK1/2 and MEK1/2 induced by Kaposi's sarcoma-associated herpesvirus (human herpesvirus 8) early during infection of target cells are essential for expression of viral genes and for establishment of infection. J Virol 79:10308–10329. doi:10.1128/JVI.79.16.10308-10329.2005. 16051824 PMC1182676

[80] Pleschka S. 2008. RNA viruses and the mitogenic Raf/MEK/ERK signal transduction cascade. Biol Chem 389:1273–1282. doi:10.1515/BC.2008.145. 18713014

[81] Albarnaz JD, De Oliveira LC, Torres AA, Palhares RM, Casteluber MC, Rodrigues CM, Cardozo PL, De Souza AM, Pacca CC, Ferreira PC, Kroon EG, Nogueira ML, Bonjardim CA. 2014. MEK/ERK activation plays a decisive role in yellow fever virus replication: implication as an antiviral therapeutic target. Antiviral Res 111:82–92. doi:10.1016/j.antiviral.2014.09.004. 25241249

[82] Martinez O, Valmas C, Basler CF. 2007. Ebola virus-like particle-induced activation of NF-κB and Erk signaling in human dendritic cells requires the glycoprotein mucin domain. Virology 364:342–354. doi:10.1016/j.virol.2007.03.020. 17434557 PMC2034500

[83] Stanley IJ, Burgess AW. 1983. Granulocyte macrophage-colony stimulating factor stimulates the synthesis of membrane and nuclear proteins in murine neutrophils. J Cell Biochem 23:241–258. doi:10.1002/jcb.240230121. 6609926

[84] Earle AJ, Kirby TJ, Fedorchak GR, Isermann P, Patel J, Iruvanti S, Moore SA, Bonne G, Wallrath LL, Lammerding J. 2020. Mutant lamins cause nuclear envelope rupture and DNA damage in skeletal muscle cells. Nat Mater 19:464–473. doi:10.1038/s41563-019-0563-5. 31844279 PMC7102937

[85] Ilinykh PA, Lubaki NM, Widen SG, Renn LA, Theisen TC, Rabin RL, Wood TG, Bukreyev A. 2015. Different temporal effects of Ebola virus VP35 and VP24 proteins on global gene expression in human dendritic cells. J Virol 89:7567–7583. doi:10.1128/JVI.00924-15. 25972536 PMC4505630

[86] Barber GN. 2011. Innate immune DNA sensing pathways: STING, AIMII and the regulation of interferon production and inflammatory responses. Curr Opin Immunol 23:10–20. doi:10.1016/j.coi.2010.12.015. 21239155 PMC3881186

[87] Hiscott J. 2007. Triggering the innate antiviral response through IRF-3 activation. J Biol Chem 282:15325–15329. doi:10.1074/jbc.R700002200. 17395583

[88] Seoane J, Le HV, Massagué J. 2002. Myc suppression of the *p21^Cip1^* Cdk inhibitor influences the outcome of the p53 response to DNA damage. Nature 419:729–734. doi:10.1038/nature01119. 12384701

[89] Ciribilli Y, Singh P, Spanel R, Inga A, Borlak J. 2015. Decoding c-Myc networks of cell cycle and apoptosis regulated genes in a transgenic mouse model of papillary lung adenocarcinomas. Oncotarget 6:31569–31592. doi:10.18632/oncotarget.5035. 26427040 PMC4741625

[90] Barsyte-Lovejoy D, Mao DY, Penn LZ. 2004. c-Myc represses the proximal promoters of GADD45a and GADD153 by a post-RNA polymerase II recruitment mechanism. Oncogene 23:3481–3486. doi:10.1038/sj.onc.1207487. 15021909

[91] Sears R, Nuckolls F, Haura E, Taya Y, Tamai K, Nevins JR. 2000. Multiple Ras-dependent phosphorylation pathways regulate Myc protein stability. Genes Dev 14:2501–2514. doi:10.1101/gad.836800. 11018017 PMC316970

[92] Wiebe MS, Jamin A. 2016. The barrier to autointegration factor: interlocking antiviral defense with genome maintenance. J Virol 90:3806–3809. doi:10.1128/JVI.00178-16. 26842478 PMC4810535

[93] Oh HS, Traktman P, Knipe DM. 2015. Barrier-to-autointegration factor 1 (BAF/BANF1) promotes association of the SETD1A histone methyltransferase with herpes simplex virus immediate-early gene promoters. mBio 6:e00345-15. doi:10.1128/mBio.00345-15. 26015494 PMC4447252

[94] Nichols RJ, Wiebe MS, Traktman P. 2006. The vaccinia-related kinases phosphorylate the N′ terminus of BAF, regulating its interaction with DNA and its retention in the nucleus. Mol Biol Cell 17:2451–2464. doi:10.1091/mbc.e05-12-1179. 16495336 PMC1446082

[95] Maciejowski J, Hatch EM. 2020. Nuclear Membrane Rupture and Its Consequences. Annu Rev Cell Dev Biol 36:85–114. doi:10.1146/annurev-cellbio-020520-120627. 32692592 PMC8191142

[96] Vidal S, El Motiam A, Seoane R, Preitakaite V, Bouzaher YH, Gómez-Medina S, San Martín C, Rodríguez D, Rejas MT, Baz-Martínez M, Barrio R, Sutherland JD, Rodríguez MS, Muñoz-Fontela C, Rivas C. 2019. Regulation of the Ebola virus VP24 protein by SUMO. J Virol 94:e01687-19 doi:10.1128/JVI.01687-19. 31597768 PMC6912094

[97] Raab M, Gentili M, de Belly H, Thiam HR, Vargas P, Jimenez AJ, Lautenschlaeger F, Voituriez R, Lennon-Duménil AM, Manel N, Piel M. 2016. ESCRT III repairs nuclear envelope ruptures during cell migration to limit DNA damage and cell death. Science 352:359–362. doi:10.1126/science.aad7611. 27013426

[98] Redwood AB, Perkins SM, Vanderwaal RP, Feng Z, Biehl KJ, Gonzalez-Suarez I, Morgado-Palacin L, Shi W, Sage J, Roti-Roti JL, Stewart CL, Zhang J, Gonzalo S. 2011. A dual role for A-type lamins in DNA double-strand break repair. Cell Cycle 10:2549–2560. doi:10.4161/cc.10.15.16531. 21701264 PMC3180193

[99] Sánchez-Aparicio MT, Ayllón J, Leo-Macias A, Wolff T, García-Sastre A. 2017. Subcellular localizations of RIG-I, TRIM25, and MAVS complexes. J Virol 91:e01155-16 doi:10.1128/JVI.01155-16. 27807226 PMC5215348

[100] García-Dorival I, Wu W, Armstrong SD, Barr JN, Carroll MW, Hewson R, Hiscox JA. 2016. Elucidation of the cellular interactome of Ebola virus nucleoprotein and identification of therapeutic targets. J Proteome Res 15:4290–4303. doi:10.1021/acs.jproteome.6b00337. 27786485

[101] Li B, Dewey CN. 2011. RSEM: accurate transcript quantification from RNA-seq data with or without a reference genome. BMC Bioinformatics 12:323. doi:10.1186/1471-2105-12-323. 21816040 PMC3163565

[102] Martin M. 2011. Cutadapt removes adapter sequences from high-throughput sequencing reads. EMBnet J 17:1–10. doi:10.14806/ej.17.1.200.

