# Supplementary figures and images for: Expression of the Ebola Virus VP24 Protein Compromises the Integrity of the Nuclear Envelope and Induces a Laminopathy-Like Cellular Phenotype
Source: mBio. 2021 Jul 6;12(4):e00972-21. doi: 10.1128/mBio.00972-21 (PMC8406168; doi:10.1128/mBio.00972-21)

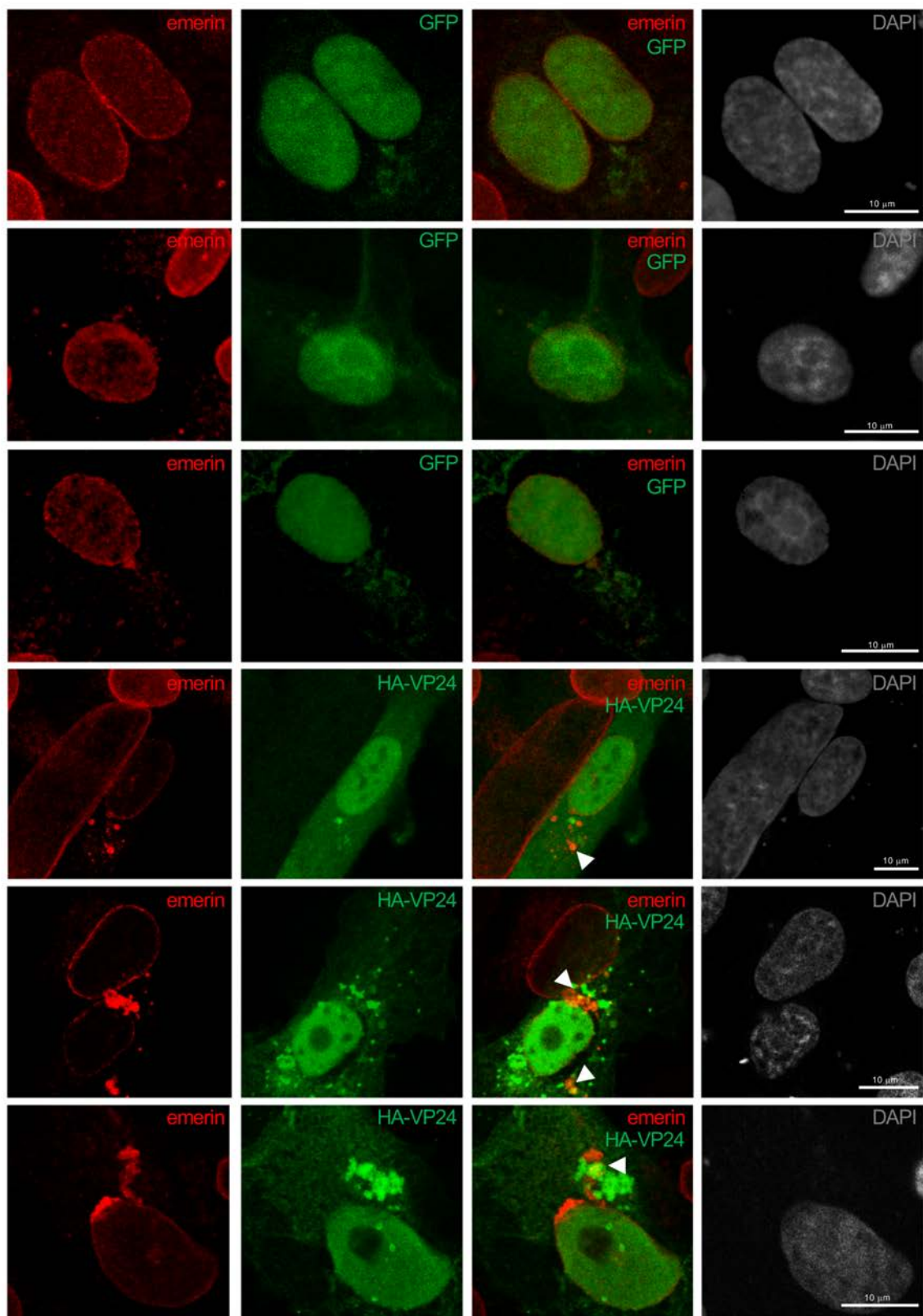

Supplement: FIG S1 [file mbio.00972-21-sf001.pdf]

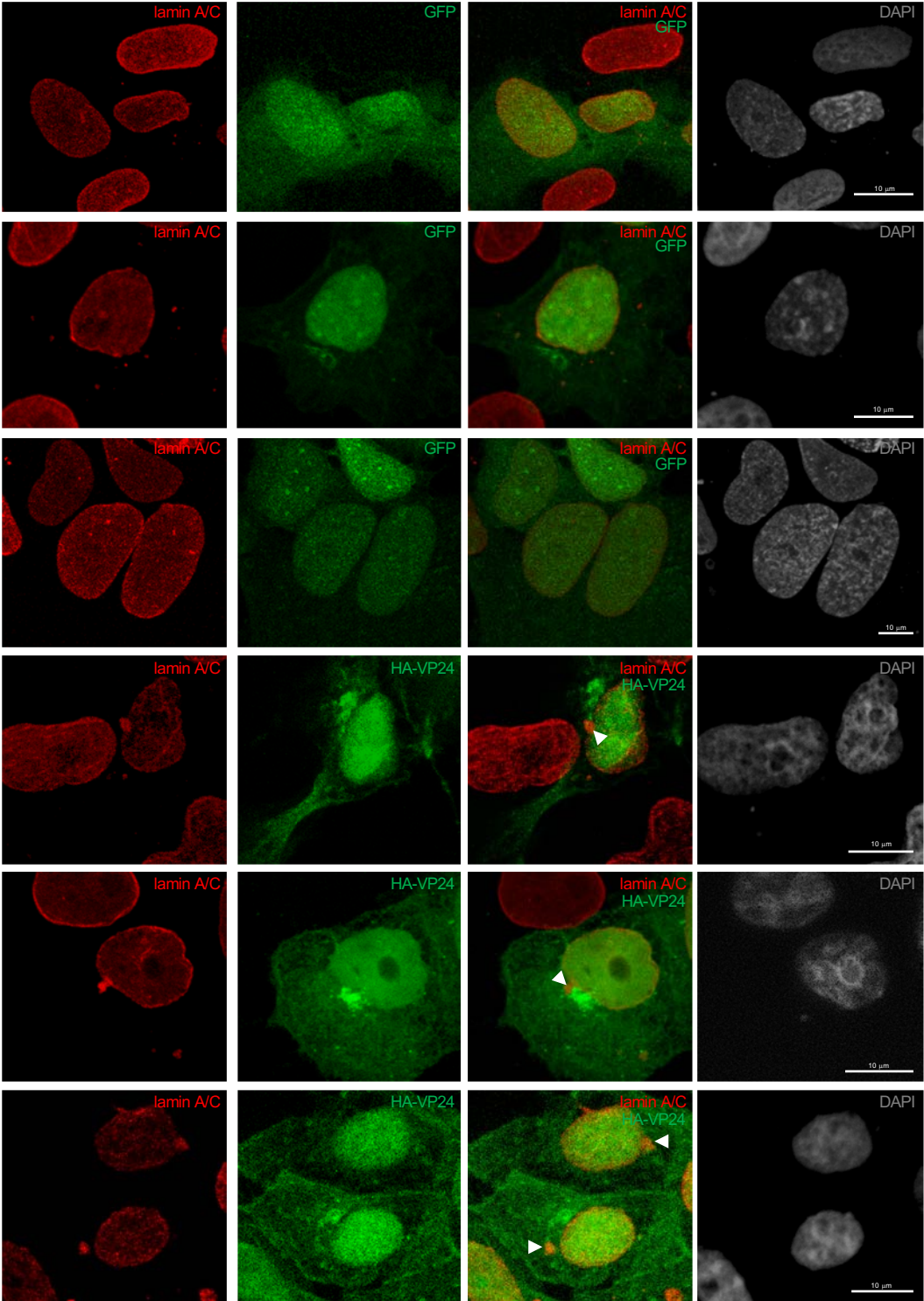

Supplement: FIG S2 [file mbio.00972-21-sf002.pdf]

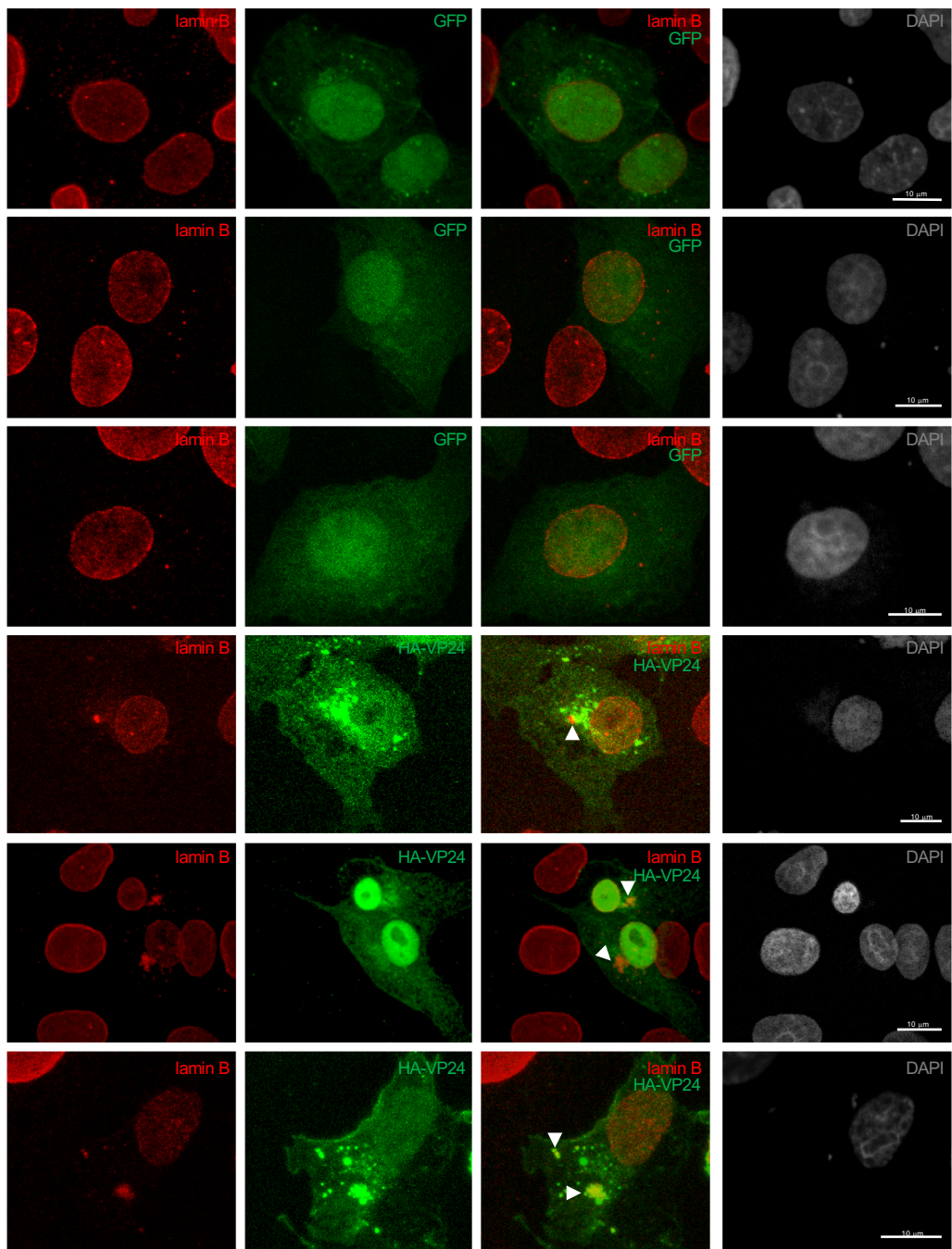

Supplement: FIG S3 [file mbio.00972-21-sf003.pdf]

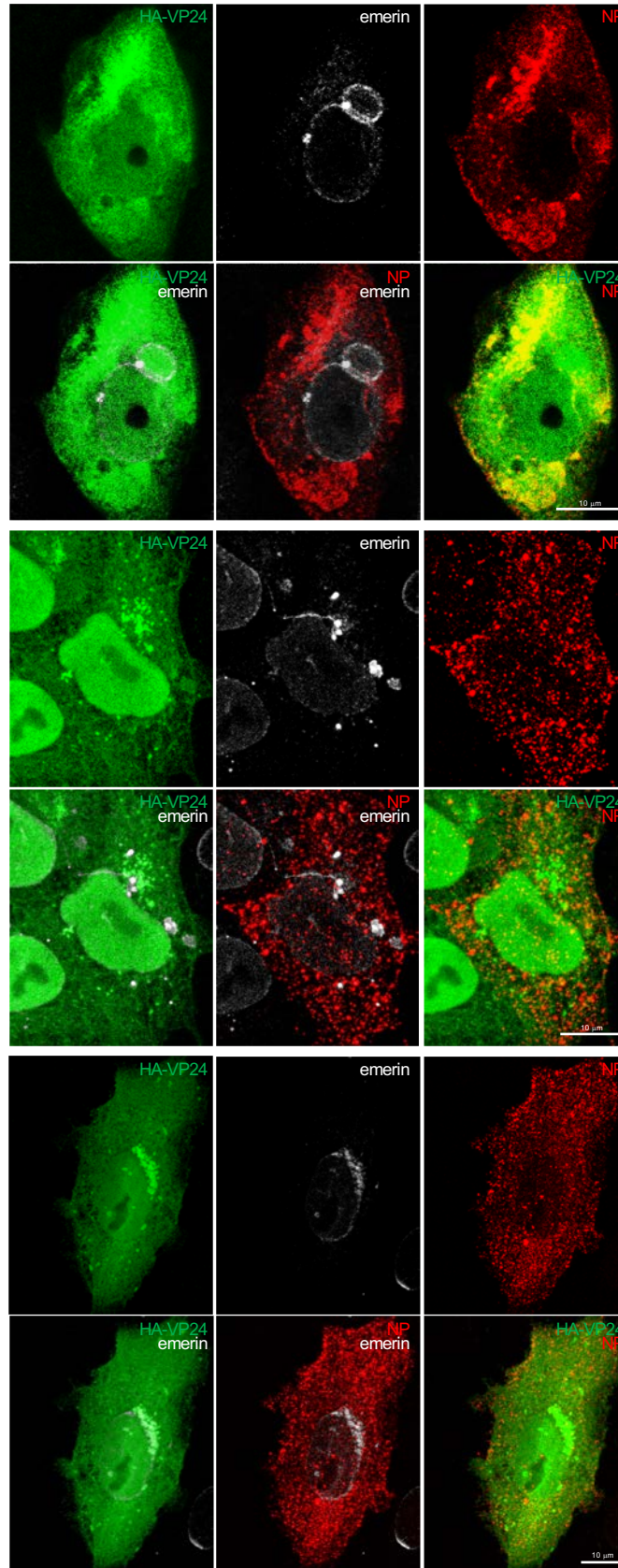

Supplement: FIG S4 [file mbio.00972-21-sf004.pdf]

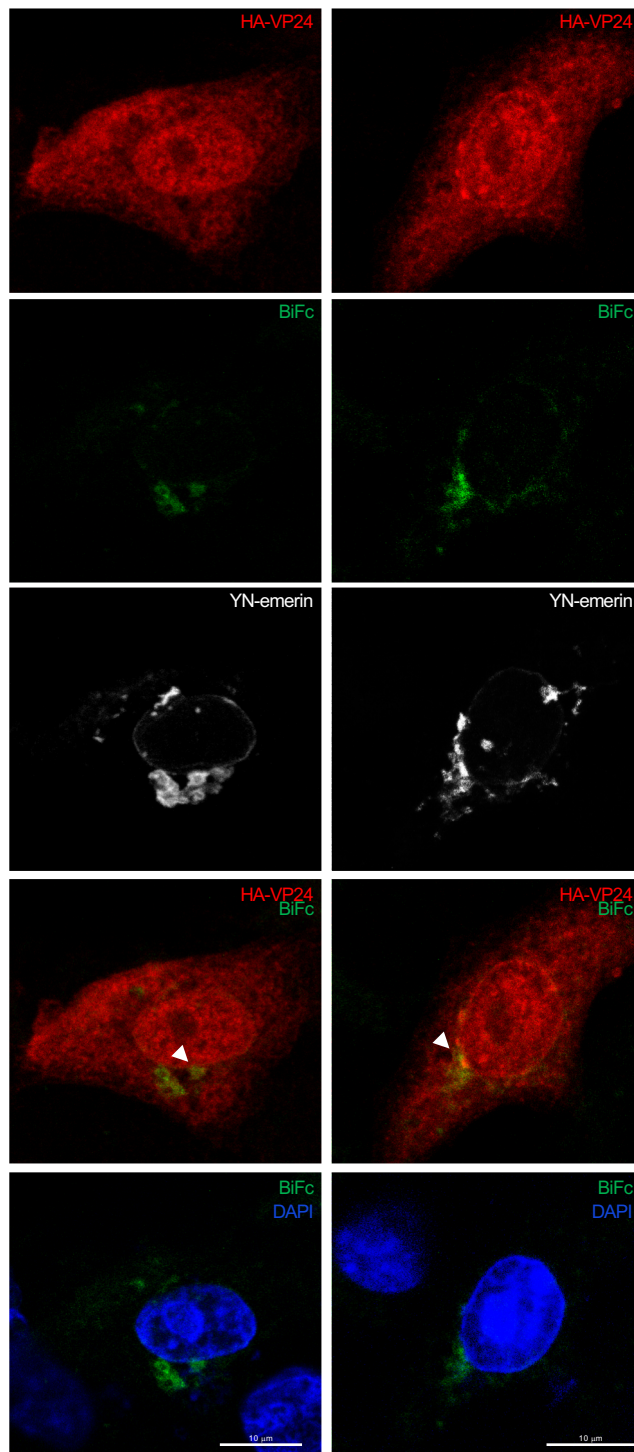

Supplement: FIG S5 [file mbio.00972-21-sf005.pdf]
